# Reinfection incidence following surgical intervention for infected aortic bypass: a meta-analysis

**DOI:** 10.1007/s10096-025-05248-9

**Published:** 2025-11-08

**Authors:** Márcio Brazuna, Marta Gonçalves-Costa, Ana Marreiros, Leonardo Araújo-Andrade, José Paulo Andrade, João Rocha-Neves

**Affiliations:** 1 Faculty of Medicine and Biomedical Sience of University of Algarve, Faro, Portugal; 2 Faculty of Medicine of University of Porto, Porto, Portugal; 3 ABC-RI, Algarve Biomedical Center Research Institute, Faro, Portugal; 4 Department of Infecciology, Unidade Local de Saúde de São João,, Porto, Portugal; 5 RISE@Health, Departament of Biomedicine – Unit of Anatomy, Faculty of Medicine of University of Porto, Porto, Portugal; 6 Department of Angiology and Vascular Surgery, Unidade Local de Saúde do Alto Ave, Guimarães, Portugal

**Keywords:** Vascular Grafting / adverse effects, Prosthesis-Related Infections, Aortic Diseases / surgery, Vascular Graft Infection, Biofilms, Nosocomial infection

## Abstract

**Background:**

Infection of vascular grafts after aortic revascularization surgery is a serious complication with high morbidity and mortality. This systematic review and meta-analysis aims to determine reinfection incidence in patients undergoing surgical intervention for infected aortic bypass grafts and identify key risk factors in the literature.

**Materials and Methods:**

This systematic review and meta-analysis followed PRISMA guidelines. Three electronic databases, PubMed/MEDLINE, Scopus, and Web of Science were used to search studies published after January 1, 2000, that assessed reinfection rates following surgical intervention for infected aortic bypass grafts. Random-effects meta-analysis was performed to calculate pooled incidence of major outcomes.

**Results:**

Our systematic review included 30 studies with a total of 2,341 patients. Overall reinfection rate was 12.7% (95% CI: 8.6%–16.9%). In terms of morbidity 34.1% had acute kidney injury, 23.8% needed amputation, and 29.4% developed acute limb ischemia. The 30-day mortality rate was 27.8% (95% CI: 13.2%–42.4%).The medical approach to treatment varied significantly, however, the majority involved total removal of the infected prosthesis. The main microorganisms isolated in primary infections were mostly Staphylococcus and Enterococcus species, with a notable representation of gram-negative bacteria.

**Conclusion:**

Reinfection rates after surgical treatment of infected aortic bypass grafts were relatively high and constitute a challenge of high clinical impact. This is further demonstrated by the high 30-day mortality rate. The significant variation in treatment approaches observed above also highlights the lack of formalized management protocols. Further studies are needed to determine the best surgical approach and patient-related risk factors to optimize outcomes in this difficult population.

**Supplementary Information:**

The online version contains supplementary material available at 10.1007/s10096-025-05248-9.

## Introduction

Vascular graft infection following aortic revascularization carries a grim prognosis: reported in‑hospital mortality ranges from 25 to 50%, and one‑year mortality can exceed 60% in some series [1, 2]. Reinfection after initial treatment of an infected aortic bypass is even worse, with mortality rates as high as 70% in certain cohorts [3]. Beyond the human toll, these infections drive substantial resource use: each case typically requires prolonged intensive‑care stays (median 14–21 days), multiple surgical interventions, and extended courses of antibiotics, leading to average per‑patient costs upwards of £59 520–£89 280 [4, 5]. Clinically, complications span from local issues—such as aortoenteric fistula (AEF) formation and graft blow‑out—to systemic sequelae including sepsis, acute kidney injury (AKI), and limb ischemia, all increasing the treatment complexity and recovery time [6, 7]. Reinfection remains a major concern, with reported recurrence rates of 10%–30% depending on the microorganisms’ virulence and host factors [8]. Traditional management still centers on broad‑spectrum antibiotics, complete graft excision, and in‑situ or extra‑anatomic bypass; however, recent reports explore less‑radical approaches such as partial graft preservation and hybrid endovascular techniques [9]. Choice of replacement material—antibiotic‑impregnated conduits, Dacron, PTFE, or cryopreserved allografts—varies widely, reflecting the lack of standardized protocols [10]. Ultimately, prognosis hinges on patient comorbidity, infecting organism virulence, and the surgical strategy employed [11].

This study evaluates reinfection rates after surgical intervention for infected aortic bypass grafts and identifies key risk factors through systematic review and meta-analysis.

## Materials and methods

This systematic review followed the Preferred Reporting Items for a Systematic Review and Meta-analysis (PRISMA) guidelines [11]. The review protocol was registered at Prospero (CRD42024605826). No ethical approval was required.

### Study identification and data extraction

Two reviewers independently screened titles and abstracts, deduplicated and assessed full‑text articles for eligibility using Rayan AI systematic review software. Discrepancies were resolved by adjudication to a third reviewer. The electronic search strategy combined Medical Subject Heading (MeSH) terms and text words—including “vascular graft infection,” “aortic bypass,” “reinfection,” “prosthetic graft,” and related synonyms—with Boolean operators; the full search strings for MEDLINE, EMBASE, and the Cochrane Library are detailed in Supplementary Table 9. Data extraction was performed in duplicate using a standardized form in Microsoft Excel (Office 365), capturing study characteristics, patient demographics, graft material, infecting organisms, reinfection rates, mortality, morbidity outcomes, and health‑system resource use. Any discrepancies in data capture were reconciled by consensus between the two reviewers. The extracted variables—including publication year, study design, sample size, patient demographics, cardiovascular comorbidities, and reinfection rates—were collated in Microsoft Excel and then imported into OpenMeta[Analyst] for statistical aggregation, including calculation of pooled reinfection rates, subgroup analyses, and generation of forest plots.

### Study selection

Studies published between January 1, 2000 and June 30, 2025 that reported reinfection rates after surgical treatment of infected aortic bypass grafts in human subjects were considered. We excluded case reports or series with fewer than five patients, reviews, editorials, conference abstracts, and any study lacking explicit reinfection outcome data. For synthesis, studies were stratified by graft material (Dacron, PTFE, antibiotic‑impregnated grafts, cryopreserved allografts), treatment strategy (complete graft excision, partial graft preservation, hybrid endovascular techniques), etiologic agent, and study design (prospective versus retrospective).

### Statistical analysis

Pooled reinfection rates and outcomes were synthesized using a random‑effects model (DerSimonian and Laird) to account for between‑study heterogeneity. Heterogeneity was assessed via the I^2^ statistic and Cochran’s Q-test. Statistical significance for all analyses was set at a two‑sided alpha level of 0.05, with 95% confidence intervals reported for summary effect estimates. Subgroup analyses were conducted by graft material, treatment strategy, and etiologic agent, while sensitivity analyses excluded studies at high risk of bias. Publication bias was evaluated using funnel plots and Egger’s regression test. Meta-regression analyses were performed to explore the influence of key study-level covariates (e.g., sample size, publication year). All quantitative syntheses, including forest plot generation, were executed in OpenMeta[Analyst].

### Publication bias

The National Heart, Lung, and Blood Institute (NHLBI) tool was used for observational cohort and cross-sectional studies (2021) studies [12]. Quality was assessed independently by two authors, with disagreements resolved through consensus. The Grading of Recommendations, Assessment, Development, and Evaluation (GRADE) approach classified articles as high, moderate, low, or very low quality [13].

## Results

### Search results

Of the 1357 screened studies, 1218 were excluded after title/abstract review. Full-text assessment was performed for 157 studies, with 115 excluded due to absence of exposure (*N* = 28) or outcome assessment (*N* = 87), and 12 unretrieved (Supplemental Fig. 1). Ultimately, thirty articles were included in this systematic review [1, 4, 5, 7, 12–37].

### Description of studies

Of 30 included studies, 29 were observational cohorts (25 retrospective) [1, 4, 5, 12–14, 16–28, 30, 33–37] and one was a case series [14] (Supplemental Table 1). Studies originated from 13 countries across North America [15–21], Europe [5, 7, 14–20, 22–24, 26, 29–32, 35–37], and Asia [22]. The analysis covered 2,341 patients undergoing aortic bypass procedures. Mean patient age ranged from 59.6 to 73 years, with standard deviations between 8.25 and 15 years. Male patients comprised 47% to 98% of study populations.

### Study quality

Supplemental Figs. 2.A and 2.B display the risk of bias. Most observational cohorts had low bias overall, except for [8, 23]. High bias was frequently associated with items D5 and D14, particularly in sample size justification and power description. Missing exposure data and follow-up losses were also concerning.

### Main findings and meta-analysis

Aortobifemoral reconstruction was the most common bypass, followed by aortoaortic, aortoiliac, aortofemoral, and extra-anatomical procedures. Ethnicity data was scarce; only one study reported a predominance of African American patients [1].

Cardiovascular risk factors were inconsistently reported; AHT and smoking were most frequent. AHT prevalence ranged from 18–93%, and smoking was up to 92%. DM occurred in 8–37% of patients. CKD and CAD were variably reported (2–48% and 1–68%, respectively). Dyslipidemia reached 71% in some studies. HF data was scarce, up to 21%, potentially due to selection bias (Supplemental Tables 2 & 3).

The mean time to infection varied widely (27–78 months). Some studies reported 7–72 early infections (≤ 4 months), but late infections (> 4 months) were more frequent (up to 149 cases). Total prosthesis removal was a frequent occurrence (up to 200 reported cases), while partial removals were less common [1, 7, 10, 15, 18, 21, 22, 24, 25]. Reconstruction configurations varied: aortobifemoral, aortoiliac, aortoaortic, and axillobifemoral were most frequent [15–17, 23, 26–31]. Graft materials were heterogeneous, including cryopreserved allografts, femoral veins, rifampicin-soaked grafts, silver-coated prostheses, PTFE, and autologous grafts (Table 1).

**Table 1:** Prosthesis Infection Characteristics, Surgical Outcomes, and Graft Configurations in Vascular Surgery Patients

**Author**	**Mean time to prothesis infection (months)**	**Early prothesis infection ** **(<4 months) **	**Late prothesis infection ** **(>4 months) **	**Total prothesis removal **	**Partial Prothesis removal**	**Reconstruction configuration**	**Graft material**
*Lesèchese G et al.*	43	NA	NA	18	10	5 Aortobifemoral; 6 Aortoaortic; 2 Aortobiiliac; 6 Aortofemoral; 8 Deep femoral artery; 2 Superior femoral artery	28 Cryopreseved allografts
*Bandyk D et al.*	NA	NA	NA	27	0	19 Aortobifemoral; 1 Thoracofemoral; 1 Femoral-femoral; 1 Axiklobifemoral	27 Rifampincin soaked
*Chiesa Ret al.*	NA	NA	NA	68	NA	40 Aortobifemoral; 12 Aortoaortic; 9 Aortofemoral; 7 Aortobiiliac	NA
*Daenes Ket al.*	59	NA	NA	49	0	44 Aortobifemoral; 3 Aortobiiliac; 2 Aorto-ilio-femoral	49 Femoral Vein
*Lavigne JP et al.*	NA	35	31	NA	NA	27 Aortobifemoral; 5 Aortoaortic; 2 Aortobiiliac; 9 Iliofemoral; 3 Axillobifemoral; 19 Femoropopliteal;	22 cryopreserved allograft; 44 unknown
*Batt M et al.*	78	NA	NA	21	6	12 Aortobifemoral	27 Dacron Silver
*Gabriel M et al.*	27	NA	NA	44	1	32 Aortobifemoral; 2 Aortofemoral; 5 Aortoiliac; 3 Femoral-femoral; 3 Axillofemoral; 1 Femoropopliteal	46 Cryopreserved allografts
*Hart J et al.*	67	NA	NA	15	15	1 Aortic Tube; 1 Aortobifemoral; 7 Aortobiiliac; 10 Axillobifemoral; 3 Axillobiprofunda; 2 Axilloprofunda	NA
*Armstrong Pet al.*	NA	9	NA	40	43	20 Aortobifemoral; 24 Extra-anatomical; 31 Ilioiliac; 12 Combined	3 Dacron Silver; 22 Rifampicin soaked; 63 PTFE
*Bisdas T et al.*	NA	NA	NA	NA	NA	NA	22 Cryopreserved allografts; 11 Dacron Silver
*Batt M et al.*	NA	NA	NA	58	15	11 Axillobifemoral; 62 in situ	26 Silver-coated; 21 Cryopreserved allograft; 8 Rifampin soaked; 6 Autogenous vein; 11 PTFe; 2 Polyester
*Legout L, et al.*	NA	49	36	41	0	NA	13 Femoral vein 12 Dacron Silver; 16 Cryopreserved allografts
*Kristofer M. Charlton-Ouw et al.*	NA	7	21	0	21	3 Aortobifemoral; 1 Axillobifemoral; 14 NAIS; 11 in situ	6 Dacron Silver; 3 Homograft; 2 PTFE; 14 Femoral vein ; 4 Unknown
*Legout L et al.*		42	34	8	76	NA	24 Arterial homograft; 12 Femoral vein
*Garot M et al.*	NA	7	18	25	0	5 Aortofemoral; 14 Aortobifemoral; 6 Aortobiiliac;	25 Cryopreserved allografts
*Heinola Iet al.*	NA	10	45	52	3	33 Aortobifemoral; 13 Aortobiiliac; 3 Aortoiliac w/ aortofemoral (contralateral); 2 Aortofemoral; 1 Aortoaortic;	55 Femoral vein
*Simmons C et al.*	NA	NA	NA	0	21	21 NAIS	22 Femoral Vein
*Bossi M et al.*	NA	NA	NA	NA	NA	3 Aortobifemoral; 7 Iliofemoral; 7 Femorotibial; 3 Femoral-femoral; 1 Femoropopliteal	21 Cryopreserved allografts
*Phang D et al.*	NA	NA	NA	49	0	34 Aortoiliac	34 Femoral vein; 18 synthetic non- specific
*Filiberto A et al.*	52	NA	NA	NA	NA	87 Aortobifemoral; 19 Aortoaortic; 11 Aortobiiliac; 13 EVAR	49 Femoral vein; 33 Cryopreserved allografts; 23 Rifampincin soaked
*Janko M, et al.*	NA	NA	NA	0	114	42 Axillobifemoral; 8 Obturator; 60 *in situ*	12 Rifampin soaked; 1 Dacron Silver; 8 Cryopreserved allografts; 12 Femoral vein; 77 Unknown
*Weiss S et al.*	NA	7	26	24	9	12 Aortobifemoral; 8 Aortobiiliac; 11 Aortofemoral	NA
*Gavali H et al.*	NA	NA	NA	NA	NA	55 *in-situ*	24 Autologous femoral vein; 17 Dacron Silver; 10 Rifampicin soaked ; 4 Autologous arterial
*Couture T et al.*	NA	51	149	200	0	NA	200 cryopreserved allografts
*Kouijzer I, et al.*	NA	NA	NA	29	0	55 Aortoaortic; 5 Iliofemoral; 8 Aortobiiliac	29 Autologous femoral vein; 2 Dacron Silver ; 37 Unknown
*Janko M et al.*	NA	NA	NA	NA	NA	NA	61 Dacron Silver 41 Femoral vein; 70 Autologous arterial
*Sixt T et al.*	NA	72	74	NA	NA	NA	NA
*Caradu C et al.*	NA	16	70	86	0	38 Aortobiiliac; 28 Aortobifemoral; 7 Aortofemoral	73 triclosan silver
*Hosaka A et al.*	NA	NA	NA	102	67	82 Aortobiiliac; 74 Axillobifemoral	47 rifampin soaked; 97 Dacron Silver; 40 PTFe; 12 femoral vein; 1 Bovine
*Weiss S et al.*	NA	NA	NA	NA	NA	NA	168 Bovine pericardium

The more frequent proximal anastomosis site was infrarenal abdominal aorta and axillary artery, while the distal anastomosis was predominantly femoral or iliac. Standard aortobifemoral grafts were the most frequently used reconstruction approach, with some studies reporting up to 40 cases [17, 23, 27]. However, the use of neo-aorta with femoral vein was also documented in several studies [1, 17, 21, 25, 26, 29, 31–33]. The obturator foramen bypass was less frequently performed, appearing in only 18 reported cases. Adjunctive surgical procedures were commonly employed, particularly debridement, muscle flap coverage, and omental flap placement to optimize graft integration and prevent reinfection. Some studies also reported complex vascular reconstructions, including celiac trunk reimplantation, renal artery bypass, mesenteric revascularization, and other extensive procedures to restore perfusion in critically ill patients (Supplemental Table 4).

Vascular graft infections were predominantly caused by gram-positive bacteria, namely *Staphylococcus aureus*, *Staphylococcus epidermidis*, and *Enterococcus spp.*, with *Methicillin-resistant Staphylococcus aureus* (MRSA) and *Vancomycin-resistant Enterococci* (VRE) also being reported. Among Gram-negative bacteria, the most common pathogens were *Escherichia coli, Pseudomonas aeruginosa* and *Klebsiella spp*. *Candida albicans* was the only fungal pathogen. Also noteworthy is a case of infection by *Coxiella burnetti*. [1, 4, 12–14, 22, 29, 34, 36]. Polymicrobial infections were frequent, often involving anaerobes like *Bacteroides spp.*. Some cases showed no microbial growth, likely due to prior antibiotic use or subpar samples (Table 2).

**Table 2:** Antibiotic Strategies, Reinfection Outcomes, and Intraoperative Complications in Vascular Graft Infections

**Author**	**Antibiotic**	**Reinfection**	**Time to reinfection**	**Reinfection micro-organism**	**Suppressive antibiotherapy**	**Reintervention**	**Intraoperative complications**
*Lesèchese G et al.*	NA	0	0	0	No	0	0
*Bandyk D et al.*	NA	2	NA	*Staphylococcus Epidermis*	NA	NA	NA
*Chiesa Ret al.*	NA	NA	NA	NA	NA	NA	NA
*Daenes Ket al.*	Vancomycin	0	0	0	No	0	0
*Lavigne JP et al.*	Vancomycin + Gentamicin + Rifampicin	9		NA	NA	NA	NA
*Batt M et al.*	Vancomycin	1	6	Non-identified in culture	NA	0	NA
*Gabriel M et al.*	NA	2	2,5	*Staphylococcus (epidermis / hominis / haemoliticus)*	NA	8	Hemorrhage; Graft rupture
*Hart J et al.*	NA	10	2		Yes	NA	NA
*Armstrong Pet al.*	NA	6	23	*MRSA; Staphylococcus; Candida albicans; Klebsiella*	No	NA	NA
*Bisdas T et al.*	NA	2	NA	NA	NA	NA	NA
*Batt M et al.*	NA	15	27.7	MRSA; Others	NA	15	NA
*Legout L, et al.*	Empirical: Beta-lactam + anti-MRSA; beta-lactam + anti-MRSA + aminoglycosides	25	NA	NA	Yes	25	Hemorrhage; Septic shock; Graft thrombosis
*Kristofer M. Charlton-Ouw et al.*	NA	7	15,08	*MSSA; MRSA; Morganella Morganii; Group D Streptococcus; Klebsiella pneumonie; Klebsiella oxytoca; Ecoli; enterococcus; araerobic gram negative.*	Yes	5	NA
*Legout L et al.*	Piperacillin-tazobactam-gentamicin; Piperacillin-tazobactam-gentamicin-teicoplanin; Piperacillin-tazobactam-gentamicin-daptomycin;	21	NA	NA	Yes	NA	NA
*Garot M et al.*	Empirical: Piperacillin-tazobactam; imipenem; third generation cephalosporin + vancomycin; daptomycin; linezolid	0	0	NA	NA	0	NA
*Heinola Iet al.*	Empirical: Cefuroxime + Vancomycin	2	2	NA	Yes	2	NA
*Simmons C et al.*	NA	3	3	*Pseudomonas Aerugionosa Bacterioides fragilis; Enterococcus faecalis; MRSA*	Yes	NA	Fasciectomy; Atrial fibrillation; Gracilis muscle flap;
*Bossi M et al.*	NA	0	0	NA	NA	NA	NA
*Phang D et al.*	NA	1	60	NA	NA	21	NA
*Filiberto A et al.*	NA	22	22	NA	Yes	NA	NA
*Janko M, et al.*	Vancomycin; Fluoroquinolones;	45	45	*Candida albicans Pseudomonas aeruginosa *	Yes	NA	NA
*Weiss S et al.*	NA	2	2	*Enterococcus spp*	Yes	14	NA
*Gavali H et al.*	NA	9	9	NA	Yes	13	NA
*Couture T et al.*	NA	17	17	NA	NA	59	Hemorrhage;
*Kouijzer I, et al.*	NA	2	2	*Coxiella burnetti; Candida albicans*	Yes	NA	Hemorrhage; Thrombosis
*Janko M et al.*	Hospital: Vancomycin; Cephalosporin Penicillin-based; Metronidazole; Fluconazole; Rifampin; Fluoroquinolone; Daptomycin;	24	24	NA	NA	NA	NA
*Sixt T et al.*	Empirical: Large-spectrum β-lactam + MRSA treatment; Ampicillin + clavulanate alone; Culture-based: Rifampicin alone or in combination; Fluoroquinolone alone or in combination;	47	47	NA	NA	NA	NA
*Caradu C et al.*	NA	6	6	*MRSA; Staphylococcus coagulase negative; Enterococcus; Pseudomonas*	Yes	4	Hemorrhage; Mesenteric Ischemia
*Hosaka A et al.*	NA	77	77	NA	NA	NA	NA
*Weiss S et al.*	NA	10	10	NA	Yes	NA	NA

The most commonly reported reinfection microorganisms included *Staphylococcus aureus* (both methicillin-sensitive (MSSA) and MRSA), *Staphylococcus epidermidis*, *Pseudomonas aeruginosa*, *Enterococcus spp.*, *Candida albicans*, *Klebsiella pneumoniae*, and *Coxiella burnetti* [1, 4, 12–14, 22, 29, 34, 36].

Antibiotic regimens varied significantly across studies, with empirical therapy frequently including broad-spectrum beta-lactams, vancomycin, aminoglycosides, rifampicin, and fluoroquinolones. The reinfection rate was heterogeneous, ranging from 0 to 77 cases per study, with time to reinfection varying widely, from 2 months to as long as 77 months (median = 14.86 months). Suppressive antibiotic therapy was prescribed in several cases, though its effectiveness in preventing reinfection remained unclear due to the lack of reporting. Reintervention was frequently necessary, with some studies documenting up to 59 cases. [19] Intraoperative complications were diverse and included hemorrhage, septic shock, thrombosis, fasciectomy, mesenteric ischemia, and muscle flap procedures (Tables 2 and 3).

**Table 3:** Renal Outcomes, Amputation Rates, and Postoperative Complications in Vascular Graft Infections

**Author**	**AKI **	**RRT (transitory)**	**RRT (persistent)**	**Amputation <30 Days (n)**	**ALI**	**Time to antibiotic treatment cessation (weeks) **	**Presence of AEF**	**Lymphatic complications ≤30 days**	**Lymphatic complications >30 days**
*Lesèchese G et al.*	0	0	0	0	0	8	0	0	0
*Bandyk D et al.*	NA	NA	NA	NA	NA	NA	NA	NA	NA
*Chiesa Ret al.*	NA	NA	NA	3	11	NA	22	NA	NA
*Daenes Ket al.*	0	0	0	0	0	0	0	0	0
*Lavigne JP et al.*	NA	NA	NA	1	1	NA	2	NA	NA
*Batt M et al.*	NA	NA	NA	NA	NA	8	0	NA	NA
*Gabriel M et al.*	NA	NA	NA	3	3	5	4	NA	NA
*Hart J et al.*	NA	NA	NA	NA	NA	NA	NA	NA	NA
*Armstrong Pet al.*	NA	NA	NA	0	0	6	NA	NA	NA
*Bisdas T et al.*	3	NA	NA	NA	NA	NA	NA	NA	NA
*Batt M et al.*	8	3	0	1	8	8.6	NA	NA	NA
*Legout L, et al.*	NA	NA	NA	NA	NA	NA	NA	NA	NA
*Kristofer M. Charlton-Ouw et al.*	NA	NA	NA	3	3	NA	2	NA	NA
*Legout L et al.*	NA	NA	NA	NA	NA	NA	NA	NA	NA
*Garot M et al.*	0	0	0	0	0	0	0	0	0
*Heinola Iet al.*	0	0	0	0	0	6	2	NA	NA
*Simmons C et al.*	3	NA	0	0	0	1-12	NA	NA	NA
*Bossi M et al.*	NA	NA	NA	NA	NA	NA	NA	NA	NA
*Phang D et al.*	NA	NA	0	0	3	NA	NA	NA	NA
*Filiberto A et al.*	NA	NA	NA	NA	NA	NA	NA	NA	NA
*Janko M, et al.*	0	0	12	29	NA	32	NA	NA	28
*Weiss S et al.*	1	1	0	0	0	12	2	NA	NA
*Gavali H et al.*	NA	4	0	2	6	12	28	NA	NA
*Couture T et al.*	NA	NA	NA	NA	NA	NA	NA	NA	NA
*Kouijzer I, et al.*	1	1	0	0	NA	6	1	1	NA
*Janko M et al.*	NA	NA	NA	NA	NA	NA	NA	NA	NA
*Sixt T et al.*	NA	NA	NA	NA	NA	5	NA	NA	NA
*Caradu C et al.*	NA	NA	NA	NA	NA	6	1	NA	NA
*Hosaka A et al.*	NA	NA	NA	NA	NA	7.85	NA	NA	NA
*Weiss S et al.*	NA	NA	NA	NA	NA	12.3	2	NA	NA

The mean operative duration varied widely among studies, ranging from 130 to 432 min. Blood loss in aortic reconstruction surgery (BLARC) was reported in some studies, with values ranging from Grade II (1,000–2,000 mL) to Grade IV (> 4,000 mL) [14, 17, 19, 20, 24, 32, 34, 35], indicating substantial variability in intraoperative bleeding control. The number of blood units transfused per procedure also varied, with reported means ranging from four to 9.4 units. Negative pressure wound therapy and wound closure by secondary intention were inconsistently reported, limiting the ability to assess their role in postoperative wound management (Supplemental Table 5).

The overall reinfection rate for prosthetic grafts in aortobifemoral bypasses specifically (n = 269) was 6.5% (95% CI: 3.4%–9.7%), with low heterogeneity (I^2^ = 14.2%, p = 0.483), these patients were included in the estimate of reinfection rates of aortic bypasses. On aortic bypasses overall, AKI occurred in 34.1% (95% CI: 12.9%–55.3%) of patients, while 13.5% (95% CI: 2.4%–24.6%) required dialysis post-graft removal. The heterogeneity for these outcomes was moderate (I^2^ = 42.9%) for dialysis and high for AKI (I^2^ = 79.3%), reflecting differences in patient populations and treatment strategies. (Supplemental Figs. 3.A-C).

Limb-related complications were also significant, with 23.8% (95% CI: 10.4%–37.2%) of patients undergoing amputation and 29.4% (95% CI: 13.6%–45.1%) experiencing ALI. Lymphatic complications were common, when reported, affecting 50.0% (95% CI: 6.2%–93.8%) of patients within 30 days and persisting beyond 30 days in 61.5% (95% CI: 47.8%–75.2%). Notably, heterogeneity was high for amputation (*I*^*2*^ = *62.2%*) and ALI (*I*^*2*^ = *52.3%*), although lymphatic complications showed no heterogeneity, its accuracy cannot be given, since only three studies reported it. (Supplemental Figs. 3.D-G).

The duration of antimicrobial treatment varied widely, with reported times ranging from one to 32 weeks. AEF, a severe complication, was reported in a few studies, reaching a maximum of 28 cases. Rates of acute kidney injury reached up to 8 cases in single series, and early amputations within 30 days were reported in as many as 29 patients [36] (Table 3).

The 30-day mortality rate was 27.8% (95% CI: 13.2%–42.4%), with substantial heterogeneity (I^2^ = 93.7%, *p* < 0.001), indicating variability across studies (Supplemental Fig. 3.H). At three months, mortality was scarce and inconsistent reported, with most studies reporting no deaths or isolated cases, reaching a maximum of 17 cases [7]. Long-term results were few and hard to summarize. Quality of life assessments were not consistently included in the analyzed studies, limiting the evaluation of long-term functional outcomes (Table 4). Long-term results were few and difficult to summarize, and quality-of-life assessments were not consistently included, limiting the evaluation of long-term functional outcomes.

Hospital length of stay varied significantly across studies, ranging from zero (in cases of intraoperative death) to 63.8 days. The duration of intensive care unit (ICU) stay was inconsistently reported, with values ranging from 2.8 to 13.7 days in studies that provided data. A limited number of studies documented hospital readmission to the ICU, with some reporting up to 19 cases [7] (Supplemental Table 6).

**Table 4:** Postoperative Mortality in Aortic Reconstruction Surgery

**Author**	**Mortality < 30 days**	**Mortality < 3 months**	**Mortality < 1 year**
*Lesèchese G et al.*	0	0	0
*Bandyk D et al.*	0	0	0
*Chiesa Ret al.*	11	NA	NA
*Daenes Ket al.*	0	0	0
*Lavigne JP et al.*	2	0	6
*Batt M et al.*	0	0	0
*Gabriel M et al.*	6	0	0
*Hart J et al.*	2	4	4
*Armstrong Pet al.*	2	0	4
*Bisdas T et al.*	NA	NA	NA
*Batt M et al.*	6	0	1
*Legout L, et al.*	12	0	0
*Kristofer M. Charlton-Ouw et al.*	2	0	1
*Legout L et al.*	0	0	14
*Garot M et al.*	0	0	0
*Heinola Iet al.*	0	1	1
*Simmons C et al.*	0	0	0
*Bossi M et al.*	NA	NA	NA
*Phang D et al.*	NA	NA	NA
*Filiberto A et al.*	NA	NA	NA
*Janko M, et al.*	28	17	0
*Weiss S et al.*	NA	0	0
*Gavali H et al.*	NA	0	0
*Couture T et al.*	NA	2	0
*Kouijzer I, et al.*	NA	0	0
*Janko M et al.*	NA	NA	NA
*Sixt T et al.*	NA	0	0
*Caradu C et al.*	NA	0	0
*Hosaka A et al.*	NA	8	4
*Weiss S et al.*	NA	2	0

**Fig. 1:**
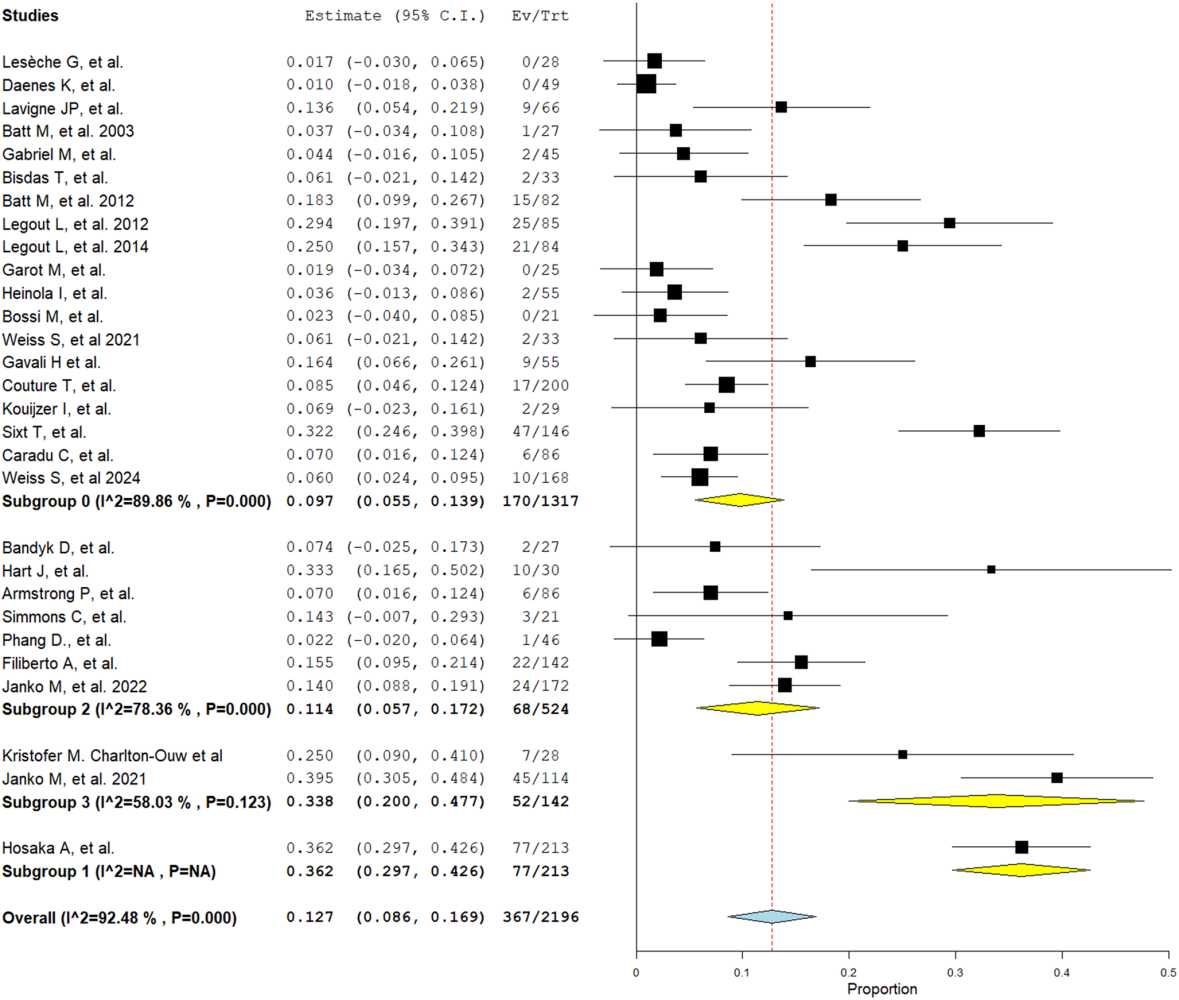
Forest plot showing reinfection incidence following surgical intervention for infected aortic bypass

The overall reinfection rate across the included studies was 12.7% (95% CI: 8.6%–16.9%), with substantial heterogeneity (I^2^ = 92.48%, *P* < 0.001). Individual study estimates varied widely, ranging from 1.7% to 39.5%. Subgroup analysis based on geographic regions indicated differences in reinfection rates: European studies reported a reinfection rate of 9.7% (95% CI: 5.5%–13.9%), North American studies had a rate of 11.4% (95% CI: 5.7%–17.1%), while the Asian study showed the highest reinfection rate at 36.2% (95% CI: 29.7%–42.6%). One of the multicentric studies had also a high reinfection rate of 33.8% (95% CI: 20.3%–47.7%) (Figure 1).

Univariate meta-regression identified age (OR: 1.02, *p* = 0.035), HF (OR: 1.01, *p* = 0.016), as significant predictors of reinfection risk after aortic bypass, dyslipidemia revealed a marginal but statistically significant (OR: 1.004, *p* = 0.044) risk as well. Other variables, including CAD, DM, smoking, CKD were not statistically significant. Geographic region did not show a strong association with reinfection risk (*p* = 0.430). (Supplemental Table 7 & 8).

## Discussion

The included studies encompassed diverse aortic bypass configurations reflecting real‑world surgical heterogeneity, with aortobifemoral being the most common. Reinfection rates varied substantially, from single‑digit percentages in small cohorts to nearly 40% in high‑risk centers [8, 22, 29, 32]. Graft materials ranged from cryopreserved allografts and rifampicin‑soaked grafts to silver‑coated prostheses and PTFE, underscoring the absence of a universally preferred conduit in infected fields and the necessity to adapt to available resources. Reinfection events occurred between 27 and 78 months postoperatively, with early infections (< 4 months) being caused by more aggressive pathogens (e.g., *S. aureus*—both MSSA and MRSA) and late infections (≥ 4 months) often being caused by fastidious and biofilm‑forming coagulase‑negative staphylococci or polymicrobial anaerobic flora [7, 14, 17, 27, 36]. This broad temporal window for infection detection emphasizes the need for prolonged vigilance well beyond discharge. Predominant pathogens and their associated antimicrobial resistances mandated the use of broad‑spectrum antimicrobials and were common and associated with higher morbidity and mortality [8, 22, 29, 32].

Meta regression identified several host factors influencing reinfection risk. Advanced age conferred modest but significant vulnerability (likely via immunosenescence and frailty) [7, 37]. Heart failure emerged as a critical predictor, probably due to impaired tissue perfusion and altered antibiotic pharmacokinetics in low output states [4, 7, 10, 37]. Dyslipidemia also reached statistical significance (OR = 1.004; 95% CI 1.000–1.010; *p* = 0.044), suggesting that lipid mediated endothelial dysfunction or inflammatory dysregulation may subtly modulate graft infection susceptibility [7, 37, 38]. Hypertension and smoking were ubiquitous comorbidities, though their independent associations varied by study [10, 19, 22].

Reinfection events per study ranged from 0 to 77, with the maximum deriving from a large multicenter cohort and representing total episodes—including repeat infections and reinterventions—rather than 77 unique patients (In Hosaka et al. [23], 213 patients contributed 77 reinfection episodes, reflecting recurrent infections in the same patient or multiple reinterventions). Time to reinfection spanned 2–77 months, but inconsistent reporting precluded a pooled median [14]. Hospital stay varied from 0 to 63.8 days; the zero day minimum likely reflects intraoperative mortality or incomplete data capture [14]. Clinically, 30 day mortality was high at 27.8% [10, 19, 23, 27], limb amputation occurred in 23.8% [20, 23, 24, 26, 28], acute limb ischemia in 29.4% [20, 23, 24, 26, 28], and acute kidney injury affected 34.1%, with 13.5% requiring dialysis [10, 21, 24, 26]. Lymphatic complications were also frequent (50.0% within 30 days, persisting in 61.5%) [7, 14, 19], underscoring the multisystem burden of these infections.

Regarding surgical management, complete graft excision with in situ reconstruction remains the gold standard [6, 12], but carries high perioperative risk. Autologous vein reconstruction—particularly the neo aortoiliac system (NAIS) using femoral vein—achieved the lowest reinfection rates (5.0–5.6%) and superior long term patency compared to synthetic materials [39, 40]. Partial graft removal, by contrast, was associated with up to 39% reinfection, attributable to retained infected foci and occult seeding—especially in the presence of aortoenteric fistulas or fungal pathogens [7, 41]. Adjunctive measures such as aggressive debridement, muscle or omental flap coverage, and complex visceral revascularizations (e.g., celiac or mesenteric bypass) were frequently employed [7, 19, 24, 26, 27, 32, 33], though direct evidence of their independent benefit remains limited by underreporting [8, 10, 16, 17, 20, 25, 30, 34].

Antibiotic regimens were heterogeneous, reflecting clinical uncertainty and delays in etiologic identification. Empirical therapy often combined broad spectrum beta lactams, vancomycin, aminoglycosides, rifampicin, and fluoroquinolones; long term suppressive therapy was reserved for patients with multidrug resistant organisms, multiple surgeries, and poor surgical candidacy [7, 10, 19, 29, 33, 41]. Suppressive strategies improved quality of life and reduced readmissions in select cohorts [14, 29, 32, 42, 43], but optimal duration and monitoring protocols remain undefined.

Our pooled reinfection (12.7%) and mortality (27.8%) rates resonate with large multicenter registries. These parallels strengthen confidence that our meta-analytic estimates reflect current surgical practice outcomes.

This review is constrained by the predominance of observational (mostly retrospective) studies, which introduces selection bias, confounding, and inconsistent definitions of reinfection, time to event, and outcome measures (Supplementary Table 8). Study heterogeneity—reflected in high I^2^ values that are inflated when event prevalences are very low or very high [44]—extends to baseline patient characteristics, designs, and methodologies, limiting direct comparability. While meta‑regression identified some patient‑level predictors, other factors (e.g., surgical technique, geographic region) showed no clear association, likely due to unmeasured confounders. Furthermore, imprecise reporting of the initial surgical procedure and limited tracking of infection sources impede causal inference. Finally, the absence of randomized trials and the reliance on data from high‑volume centers may reduce generalizability to broader clinical settings and underscore the challenges of studying this low‑incidence condition.

Establishing prospective multicenter registries with standardized definitions of graft infection, uniform data collection templates, and predefined follow-up intervals to enable robust surveillance and comparative effectiveness research is necessary. Coordinated randomized trials—where feasible—should evaluate complete versus partial graft explantation and open versus endovascular strategies in defined risk strata, thereby informing evidence-based guidelines for this challenging clinical entity.

## Conclusion

This systematic review, which pooled data from 2,341 patients, found a reinfection rate of 12.7% and a 30‑day mortality of 27.8% following surgical treatment of aortic graft infections. Advanced age, heart failure, and dyslipidemia emerged as independent predictors of reinfection, underscoring the need for targeted pre‑ and perioperative optimization in these high‑risk groups. Wide variability in conduits (autologous vein vs. cryopreserved allograft vs. PTFE or antibiotic‑impregnated prostheses) and in approaches (complete excision, partial preservation, endovascular rescue) highlights the absence of consensus on graft selection; future guidelines should articulate evidence‑based criteria for conduit choice, taking into account infection severity, patient comorbidity, and anatomical considerations. Likewise, heterogeneity in antibiotic regimens and suppressive strategies points to the need for standardized protocols—specifying agent selection, duration of therapy, and monitoring schedules—to maximize microbial eradication while minimizing resistance and toxicity. Finally, our findings reinforce the value of multidisciplinary care teams and the establishment of prospective multicenter registries to harmonize definitions, collect granular data on surgical and medical management, and ultimately drive guideline development aimed at reducing the substantial morbidity and mortality associated with aortic graft reinfection.

## Supplementary Information

Below is the link to the electronic supplementary material.

Supplementary Tables (PDF 292 KB)

Supplementary Figure Captions (PDF 106 KB)

Supplemental Figure 1 (PDF 74.2 KB)

Supplemental Figure 2A (PNG 266 KB)

High Resolution Image (TIF 11.2 MB)

Supplemental Figure 2B (PNG 266 KB)

High Resolution Image (TIF 466 KB)

Supplemental Figure 3A (PNG 266 KB)

High Resolution Image (TIF 331 MB)

Supplemental Figure 3B (PNG 266 KB)

High Resolution Image (TIF 379 KB)

Supplemental Figure 3C (PNG 266 KB)

High Resolution Image (TIF 325 KB)

Supplemental Figure 3D (PNG 266 KB)

High Resolution Image (TIF 448 KB)

Supplemental Figure 3E (PNG 266 KB)

High Resolution Image (TIF 387 KB)

Supplemental Figure 3F (PNG 266 KB)

High Resolution Image (TIF 197 KB)

Supplemental Figure 3G (PNG 266 KB)

High Resolution Image (TIF 218 KB)

Supplemental Figure 3H (PNG 266 KB)

High Resolution Image (TIF 397 KB)

## Data Availability

No datasets were generated or analysed during the current study.
