# Supplementary material for: Reinfection incidence following surgical intervention for infected aortic bypass: a meta-analysis
Source: Eur J Clin Microbiol Infect Dis. 2025 Nov 8;45(2):351–62. doi: 10.1007/s10096-025-05248-9 (PMC12987887; doi:10.1007/s10096-025-05248-9)
Supplement: Supplementary file 1 — Supplementary Tables (PDF 292 KB) [file 10096_2025_5248_MOESM1_ESM.pdf]

Supplemental Table 1 – Summary of Vascular Surgery Studies: Study Designs, Centers, and Geographic Distribution.

**Author**	**Journal**	**Publication year**	**Study design**	**Study Center**	**Country**	**Continent**
Lesèchese G et al.	Journal of Vascular Surgery	2001	Prospective Cohort	Hôpital Beaujon, Clichy	France	Europe
Bandyk D et al.	Journal of Surgical Research	2001	Retrospetive Cohort	Multicenter	USA	North America
Chiesa Ret al.	Acta Chirurgica Belgica	2002	Retrospetive Cohort	Multicenter	Italy	Europe
Daenes Ket al.	European Journal of Vascular and Endovascular Surgery	2003	Retrospetive Cohort	University Hospital Gasthuisberg	Belgium	Europe

Lavigne JP et al.	European Journal of Vascular and Endovascular Surgery	2003	Retrospetive Cohort	Cardiovascular Surgery Department, University Hospital of Lie¡ge	Belgium	Europe
Batt M et al.	Journal of Vascular Surgery	2003	Prospective Cohort	Multicenter	Fraca, Italy, Spain	Europe
Gabriel M et al.	European Journal of Vascular and Endovascular Surgery	2004	Retrospetive Cohort	Department of Vascular Surgery, Medical University of Poznań	Poland	Europe
Hart J et al.	Annals of Vascular Surgery	2005	Retrospetive Cohort	Multicenter	USA	North America

Armstrong Pet al.	Journal of Vascular Surgery	2007	Retrospetive Cohort	Division of Vascular and Endovascular Surgery, University of South Florida School of Medicine	USA	North America
Bisdas T et al.	Journal of Vascular Surgery	2010	Retrospetive Cohort	Hannover Medical School	Germany	Europe
Batt M et al.	Journal of Vascular Surgery	2012	Retrospetive Cohort	Multicenter	France	Europe
Legout L, et al.	Clinical Microbiology and Infection	2012	Prospective Cohort	Infectious Diseases e Vascular Surgery Departments dos hospitais de Lille e Tourcoing	France	Europe

Kristofer M. Charlton-Ouw et al.	Journal of Vascular Surgery	2014	Retrospetive Cohort	Multicenter	Multiple	Multicontinent
Legout L et al.	BMC Infectious Diseases	2014	Prospective Cohort	Infectious Diseases e Vascular Surgery Departments dos hospitais de Lille e Tourcoing	France	Europe
Garot M et al.	BMC Infectious Diseases	2014	Retrospetive Cohort	Hôpital Chatiliez	France	Europe
Heinola Iet al.	European Journal of Vascular and Endovascular Surgery	2016	Retrospetive Cohort	Helsinky University Hospital	Finland	Europe

Simmons C et al.	Journal of Vascular Surgery	2017	Retrospetive Cohort	University of Arkansas for Medical Sciences	USA	North America
Bossi M et al.	Annals of Vascular Surgery	2017	Retrospetive Cohort	Circolo University Teaching Hospital	Italy	Europe
Phang D et al.	Annals of Vascular Surgery	2018	Retrospetive Cohort	University of Cincinnati Medical Center; University of Arkansas for Medical Sciences	USA	North America
Filiberto A et al.	Elsevier	2021	Retrospetive Cohort	Division of Vascular Surgery & Endovascular Therapy, University of Florida	USA	North America

Janko M, et al.	Annals of Vascular Surgery	2021	Retrospetive Cohort	Multicenter	Multiple	Multicontinent
Weiss S et al.	Journal of Vascular Surgery	2021	Retrospetive Cohort	Bern University	Switzerland	Europe
Gavali H et al.	European Journal of Vascular and Endovascular Surgery	2021	Retrospetive Cohort	Multicenter	Sweden	Europe
Couture T et al.	European Journal of Vascular and Endovascular Surgery	2021	Retrospetive Cohort	Hospital Pitié-Salpêtrière	France	Europe
Kouijzer I, et al.	Annals of Vascular Surgery	2022	Retrospetive Cohort	Radboud University Medical Center	Netherlands	Europe

Janko M et al.	Journal of Vascular Surgery	2022	Retrospetive Cohort	Multicenter	USA	North America
Sixt T et al.	Open Forum Infectious Diseases	2022	Retrospetive Cohort	Dijon-Bourgogne University Hospital	France	Europe
Caradu C et al.	Journal of Vascular Surgery	2023	Retrospetive Cohort	Bordeaux University Hospital	France	Europe
Hosaka A et al.	European Journal of Vascular and Endovascular Surgery	2023	Retrospetive Cohort	Multicenter	Japan	Asia
Weiss S et al.	European Journal of Vascular and Endovascular Surgery	2024	Retrospetive Cohort	Multicenter	Multiple	Europe

Supplemental Table 2 - Patient Characteristics and Original Surgical Configurations

**Author**	**Sample size**	**Original Configuration**	**Mean Age**	**Standard Deviation**	**Male**	**Race**
Lesèchese G et al.	28	NA	64	NA	27	NA
Bandyk D et al.	27	19 Aortobifemoral; 1 Thoracofemoral; 1 Femoral-femoral; 1 Axillobifemoral	68	NA	22	NA
Chiesa Ret al.	68	68 Aortoaortic	65	NA	NA	NA
Daenes Ket al.	49	NA	65	NA	45	NA
Lavigne JP et al.	66	NA	63.75	8,25	60	NA

Batt M et al.	27	4 Aortoaortic; 4 Aortobiiliac; 11 Aortobifemoral; 2 Aortofemoral ; 6 Iliofemoral;	69	NA	25	NA
Gabriel M et al.	45	32 Aortobifemoral; 2 Aortofemoral; 5 Aortoiliac; 3 Femoral-femoral; 3 Axillofemoral; 1 Femoropopliteal	61	NA	38	NA
Hart J et al.	30	NA	70	NA	27	NA
Armstrong Pet al.	86	24 Aortobifemoral; 19 Extra-anatomical; 31 Ilioiliac; 12 Combined	NA	NA	47	NA
Bisdas T et al.	33	6 Aortobifemoral; 10 Aortic Tube; 11 Aortobiiliac; 6 Aortobiprofunda	64,5	12	32	NA
Batt M et al.	82	55 Aortofemoral; 9 Aortoaortic; 18 Aortoiliac;	69,43	NA	79	NA
Legout L, et al.	85	NA	67,8	12,2	74	NA

Kristofer M. Charlton-Ouw et al.	28	21 Aortobifemoral; 5 Aortoiliac; 2 EVAR	69	12,2	19	Caucasian: n=4<br>African American: n =21<br>Other: n=2
Legout L et al.	84	47 Femoral-femoral; 37 Femoropopliteal	64,5	11,4	72	NA
Garot M et al.	25	5 Aortofemoral; 14 Aortobifemoral; 6 Aortobiiliac	67	8,4	25	NA
Heinola Iet al.	55	NA	67	15	13	NA
Simmons C et al.	21	3 Aortobifemoral; 18 Aortofemoral	61,4	12,2	19	NA
Bossi M et al.	21	4 Aortobifemoral; 8 Femoropopliteal; 3 Femoral-femoral; 3 Iliofemoral; 3 Endarterectomy	NA	NA	34	NA

Phang D et al.	46	39 Aortobifemoral	59,6	11,5	98	NA
Filiberto A et al.	142	87 Aortobifemoral; 19 Aortoaortic; 11 Aortobiiliac; 13 EVAR	66,6	11	79	NA
Janko M, et al.	114	88 Aortobifemoral; 18 Aortobiiliac; 6 Aortic Tube	66,1	NA	30	NA
Weiss S et al.	33	33 Aortoiliac	67	NA	30	NA
Gavali H et al.	55	NA	70,4	8,6	44	NA
Couture T et al.	200	139 Aortofemoral; 36 Aortoiliac; 17 Aortoaortic; 4 Iliofemoral; 4 Femoral-femoral	64,2	9,4	184	NA
Kouijzer I, et al.	29	2 Aortofemoral; 5 Aortic Tube; 4 Aortobiiliac; 12 EVAR	69	9	27	NA

Janko M et al.	172	172 in situ with unknown configuration	68	NA	128	NA
Sixt T et al.	146	21 Aortobifemoral	68	NA	144	NA
Caradu C et al.	86	21 Aortobifemoral; 2 Aortoiliac; 1 Aortic Tube; 3 Femoral-femoral; 1 Axillobifemoral; 32 Native; 26 EVAR	69	NA	74	NA
Hosaka A et al.	213	213 Aortoiliac	73	NA	188	NA
Weiss S et al.	168	NA	67	11	130	NA

Supplemental Table 3 – Cardiovascular risk factors and comorbidities.

**Author**	**HTA**	**DM**	**Tabagism**	**Dyslipidemia**	**CKD**	**CAD**	**HF**
Lesèchese G et al.	NA	NA	NA	NA	NA	NA	NA
Bandyk D et al.	NA	NA	NA	NA	NA	NA	NA
Chiesa Ret al.	NA	NA	NA	NA	NA	NA	NA
Daenes Ket al.	NA	NA	NA	NA	NA	NA	NA
Lavigne JP et al.	NA	NA	NA	NA	NA	NA	NA
Batt M et al.	11	4	24	5	3	10	NA
Gabriel M et al.	8	4	NA	NA	NA	10	NA
Hart J et al.	NA	NA	NA	NA	NA	NA	NA

Armstrong Pet al.	75	32	55	NA	2	59	NA
Bisdas T et al.	21	10	NA	9	NA	14	NA
Batt M et al.	56	11	56	NA	5	40	NA
Legout L, et al.	66	24	NA	NA	7	44	NA
Kristofer M. Charlton-Ouw et al.	24	6	26	15	8	14	NA
Legout L et al.	NA	25	NA	NA	24	48	NA
Garot M et al.	18	4	NA	NA	12	16	NA
Heinola Iet al.	40	23	20	NA	NA	20	NA
Simmons C et al.	NA	NA	NA	NA	3	NA	NA

Bossi M et al.	18	7	NA	12	7	12	NA
Phang D et al.	43	15	41	33	12	36	10
Filiberto A et al.	119	39	103	79	NA	59	13
Janko M, et al.	98	23	84	NA	NA	25	19
Weiss S et al.	30	8	3	19	10	16	6
Gavali H et al.	33	5	18	NA	7	12	3
Couture T et al.	122	29	181	98	33	54	NA
Kouijzer I, et al.	NA	NA	NA	NA	NA	NA	NA
Janko M et al.	146	50	132	NA	NA	2	19
Sixt T et al.	NA	33	NA	NA	22	NA	29

Caradu C et al.	61	18	64	47	11	21	NA
Hosaka A et al.	158	40	130	NA	12	63	NA
Weiss S et al.	NA	37	NA	NA	47	65	NA

Supplemental Table 4 - Surgical Configurations, Graft Types, and Adjunctive Procedures in Vascular Graft Interventions

**Author**	**Proximal Anastomosis**	**Distal Anastomosis**	**Regular Aortobifemoral graft**	**Neo-aorta with femoral vein**	**Obturator Foramen Bypass**	**Adjunctive surgical procedures**
Lesèchese G et al.	NA	Femoral	5	0	0	NA
Bandyk D et al.	NA	NA	19	NA	NA	NA
Chiesa Ret al.	NA	NA	40	NA	NA	Debridement; Muscle flap coverage; Omental flap
Daenes Ket al.	Infrarrenal AA	Femoral; Iliac	0	49	NA	Muscle flap coverage; Omental flap
Lavigne JP et al.	NA	NA	NA	NA	NA	NA

Batt M et al.	NA	NA	11	0	NA	Debridement; Muscle flap coverage; Omental flap
Gabriel M et al.	NA	NA	32	0	0	Debridement; Omental flap
Hart J et al.	NA	NA	1	0	0	Muscle flap coverage
Armstrong Pet al.	NA	NA	24	7	NA	Debridement; Muscle flap coverage;
Bisdas T et al.	NA	NA	NA	NA	NA	NA
Batt M et al.	Axilar	Femoral	NA	NA	NA	Duodenorrhaphy; Segmental duodenal ressection;<br>End-to-end duodenal anastomosis;<br>Gastrojejunostomy.

Legout L, et al.	NA	NA	28	13	0	Debridement; Muscle flap coverage; Omental flap
Kristofer M. Charlton-Ouw et al.	NA	Femoral	4	3	0	NA
Legout L et al.	Infrarrenal AA	Femoral; Popliteal	NA	NA	NA	NA
Garot M et al.	NA	NA	NA	NA	NA	Omental flap
Heinola Iet al.	Infrarrenal AA	Femoral	0	55	0	Muscle flap coverage
Simmons C et al.	Infrarrenal AA	Femoral	0	21	0	Muscle flap coverage

Bossi M et al.	NA	NA	3	NA	NA	NA
Phang D et al.	NA	Femoral; Iliac	0	34	18	NA
Filiberto A et al.	Infrarrenal AA; Axilar	Femoral	38	42	0	NA
Janko M, et al.	NA	Femoral	NA	12	8	Muscle flap coverage ; Omental flap
Weiss S et al.	NA	NA	12	NA	NA	Omental flap; Xenopericardial patch
Gavali H et al.	NA	NA	31	24	0	NA
Couture T et al.	Infrarrenal AA	Femoral; Iliac	NA	NA	NA	Debridement; Muscle flap coverage; Omental flap

Kouijzer I, et al.	Infrarrenal AA	Femoral; Iliac	0	29	NA	NA
Janko M et al.	NA	NA	NA	41	NA	Muscle flap coverage
Sixt T et al.	NA	NA	21	NA	NA	NA

Caradu C et al.	Supra-renal; Celiac	Aorticbifemoral Aorticbiiliac	26	NA	NA	Omental flap; Temporary shunt to celiac trunk; Celiac trunk reimplantation/bypass; Initial thoracoabdominal bypass; Temporary shunts to SMA; SMA reimplantation/bypass; Initial thoracoaortic bypass; Cold perfusion of renal arteries; Inferior mesenteric artery reimplantation; 6 renal artery reimplantation/bypass/ligatio

						n; 4 hypogastric artery bypass/ligation; 8 Fogarty catheter thrombectomy; 2 splenectomy; 1 nephrectomy; 2 colectomy; 3 vena cava repair; 3 lumbar arthrodesis; 3 ureterovesical repair; 3 ureteral stents; artery reimplantation; 6 renal artery reimplantation/bypass/ligation; 4 hypogastric artery bypass/ligation; 8 Fogarty catheter thrombectomy; 2 splenectomy; 1 nephrectomy; 2 colectomy; 3 vena cava repair; 3 lumbar arthrodesis;

						3 ureterovesical repair; 3 ureteral stents

Hosaka A et al.	NA	NA	NA	NA	NA	NA
Weiss S et al.	NA	NA	NA	NA	NA	NA

Supplemental Table 5 - Operative Duration, Blood Loss, and Wound Management in Aortic Reconstruction Surgery.

**Author**	**Mean operation duration (minutes)**	**BLARC (Blood Loss in Aortic Reconstruction Surgery)**	**Blood Units**	**Negative pressure Wound Therapy**	**Wound Closure second intention**
Lesèchese G et al.	130	NA	NA	NA	NA
Bandyk D et al.	NA	NA	NA	NA	NA
Chiesa Ret al.	NA	NA	NA	NA	NA
Daenes Ket al.	360	NA	NA	NA	NA
Lavigne JP et al.	NA	NA	NA	NA	NA

Batt M et al.	NA	NA	NA	NA	NA
Gabriel M et al.	NA	NA	NA	NA	NA
Hart J et al.	NA	NA	NA	NA	NA
Armstrong Pet al.	NA	NA	NA	NA	NA
Bisdas T et al.	243,3	NA	7.3	NA	NA
Batt M et al.	NA	III	NA	NA	NA
Legout L, et al.	NA	NA	NA	NA	NA
Kristofer M. Charlton-Ouw et al.	NA	NA	9.4	NA	NA
Legout L et al.	NA	NA	NA	NA	NA
Garot M et al.	270	NA	5.7	NA	NA

Heinola Iet al.	432	IV	NA	NA	NA
Simmons C et al.	NA	NA	NA	NA	NA
Bossi M et al.	240	II	NA	NA	NA
Phang D et al.	375	II	NA	NA	NA
Filiberto A et al.	NA	II	5	NA	NA
Janko M, et al.	NA	NA	NA	NA	NA
Weiss S et al.	420	NA	NA	NA	NA
Gavali H et al.	NA	NA	NA	NA	NA
Couture T et al.	NA	III	8.6	NA	NA
Kouijzer I, et al.	377	II	NA	NA	NA

Janko M et al.	360	II	NA	NA	NA
Sixt T et al.	NA	NA	NA	NA	NA
Caradu C et al.	279.9	III	4	NA	NA
Hosaka A et al.	NA	NA	NA	NA	NA
Weiss S et al.	NA	NA	NA	NA	NA

Supplemental Table 6 - Postoperative Hospital and ICU Stay in Aortic Reconstruction Surgery.

**Author**	**Hospital Stay (days)**	**Mean ICU Stay duration (days)**	**Readmission to ICU (n)**
Lesèchese G et al.	0	0	0
Bandyk D et al.	NA	NA	NA

Chiesa Ret al.	NA	NA	NA
Daenes Ket al.	0	0	0
Lavigne JP et al.	63.8	NA	NA
Batt M et al.	NA	NA	NA
Gabriel M et al.	18	NA	NA
Hart J et al.	NA	NA	NA
Armstrong Pet al.	17	NA	NA
Bisdas T et al.	21.3	NA	NA
Batt M et al.	23	NA	NA
Legout L, et al.	NA	NA	12

Kristofer M. Charlton-Ouw et al.	25	13.7	NA
Legout L et al.	NA	NA	NA
Garot M et al.	NA	NA	NA
Heinola Iet al.	NA	NA	NA
Simmons C et al.	5.6	2.8	NA
Bossi M et al.	32	3	NA
Phang D et al.	11,5	NA	NA
Filiberto A et al.	23	NA	NA
Janko M, et al.	NA	NA	19
Weiss S et al.	20	NA	NA

Gavali H et al.	24	3	NA
Couture T et al.	NA	NA	NA
Kouijzer I, et al.	NA	3	NA
Janko M et al.	NA	NA	NA
Sixt T et al.	NA	NA	NA
Caradu C et al.	24	11	NA
Hosaka A et al.	NA	NA	NA
Weiss S et al.	NA	NA	NA

Supplemental Table 7

Covariate	Odds Ratio (OR)	Lower Bound	Upper Bound	Std. Error	p-Value
Age	1.02	1.0	1.04	0.01	**0.035**
CAD	1.0	0.99	1.01	0.002	0.521
Partial Removal	1.0	1.0	1.0	0.001	**0.009**
Total Removal	1.0	0.99	1.01	0.001	0.835
Vein Reconstruction	0.99	0.98	1.01	0.003	0.421
NAIS Procedure	0.99	0.98	1.01	0.002	0.298
Male	1.0	1.0	1.01	0.001	**0.005**
Cryopreserved Allograft	1.0	1.0	1.0	0.001	**0.011**
Obturator Bypass	0.99	0.98	1.01	0.008	0.712
Blood Units Transfused	1.0	0.98	1.02	0.011	0.899
AHT	1.0	1.0	1.01	0.001	**0.002**
Dyslipidemia	1.004	1.0	1.01	0.001	**0.044**

CKD	1.0	0.99	1.01	0.002	0.948
HF	1.01	1.0	1.02	0.004	**0.016**
DM	1.0	1.0	1.01	0.002	**0.051**
Continent	1.02	0.97	1.06	0.02	0.430
Smoking	1.0	1.0	1.01	0.001	0.192

Supplemental Table 8

Covariate	Odds Ratio (OR)	Lower Bound	Upper Bound	Std. Error	p-Value
AKI	1.02	1.02	1.03	0.003	< 0.001
RRT Temporary	1.01	0.95	1.07	0.031	0.699
RRT – definitive	NA	NA	NA	NA	NA
Amputation	1.03	1.02	1.04	0.005	< 0.001
ALI	1.01	1.01	1.02	0.002	< 0.001
Mortality 3 Months	1.02	0.99	1.05	0.013	0.057
Mortality 1 Year	1.02	1.01	1.03	0.005	0.002

Supplemental Table 9 – Search query – key words

**Bibliographic source**	**Search term**	**No of reports**
Pubmed/Medline	(((aorta OR aortic))<br>OR<br>(aorto-iliac OR iliac OR aortoiliac OR aortofemoral OR aorto-femoral OR aortobifemoral OR aorto-bifemoral))<br>AND<br>((infection[ti] OR infected [ti] OR infections[ti])<br>AND	863

	(prosthesis[ti] OR prosthetic[ti] OR graft[ti] OR grafts[ti] OR implant[ti] OR implants[ti] OR “aortofemoral bypass”[ti] OR “aortobifemoral bypass”[ti] OR bypass[ti]))	
Embase	(aorta OR aortic OR aorto-iliac OR iliac OR aortoiliac OR aortofemoral OR aorto-femoral OR aortobifemoral OR aorto-bifemoral)<br>AND<br>(infection OR infected OR infections)<br>AND	574

	(prosthesis OR prosthetic OR graft OR grafts OR implant OR implants OR “aortofemoral bypass” OR “aortobifemoral bypass” OR bypass)	
Scopus	(((aorta OR aortic)) OR (aorto-iliac OR iliac OR aortoiliac OR aortofemoral OR aorto-femoral OR aortobifemoral OR aorto-bifemoral)) AND ((infection[ti] OR infected [ti] OR infections[ti]) AND	5

	(prosthesis[ti] OR prosthetic[ti] OR graft[ti] OR grafts[ti] OR implant[ti] OR implants[ti] OR “aortofemoral bypass”[ti] OR “aortobifemoral bypass”[ti] OR bypass[ti]))	
