# Supplementary material for: Reinfection incidence following surgical intervention for infected aortic bypass: a meta-analysis
Source: Eur J Clin Microbiol Infect Dis. 2025 Nov 8;45(2):351–62. doi: 10.1007/s10096-025-05248-9 (PMC12987887; doi:10.1007/s10096-025-05248-9)
Supplement: Supplementary file 2 — Supplementary Figure Captions (PDF 106 KB) [file 10096_2025_5248_MOESM2_ESM.pdf]

Supplemental Figure 1: PRISMA flow diagram illustrating the study identification and selection process.

Supplemental Figure 2.A: Risk of bias assessment for all cohort studies included in the systematic review, presented by individual article.

Supplemental Figure 2.B: Risk of bias assessment for all cohort studies included in the systematic review, presented by individual answer.

Supplemental Figure 3.A: Overall reinfection rate for prosthetic grafts in aortic reconstruction with femoral surgery.

Supplemental Figure 3.B: Overall Acute Kidney Injury rate.

Supplemental Figure 3.C: Overall Renal placement therapy rate.

Supplemental Figure 3.D: Overall amputation rate.

Supplemental Figure 3.E: Overall Acute Limb Ischemia rate.

Supplemental Figure 3.F: Overall Lymphatic complications <30 days rate.

Supplemental Figure 3.G: Overall Lymphatic complications >30 days rate.

Supplemental Figure 3.H: Overall 30-day mortality rate.
