# Supplementary material for: Reinfection incidence following surgical intervention for infected aortic bypass: a meta-analysis
Source: Eur J Clin Microbiol Infect Dis. 2025 Nov 8;45(2):351–62. doi: 10.1007/s10096-025-05248-9 (PMC12987887; doi:10.1007/s10096-025-05248-9)
Supplement: Supplementary file 3 — Supplemental Figure 1 (PDF 74.2 KB) [file 10096_2025_5248_MOESM3_ESM.pdf]

PRISMA 2020 flow diagram for new systematic reviews which included searches of databases and registers only

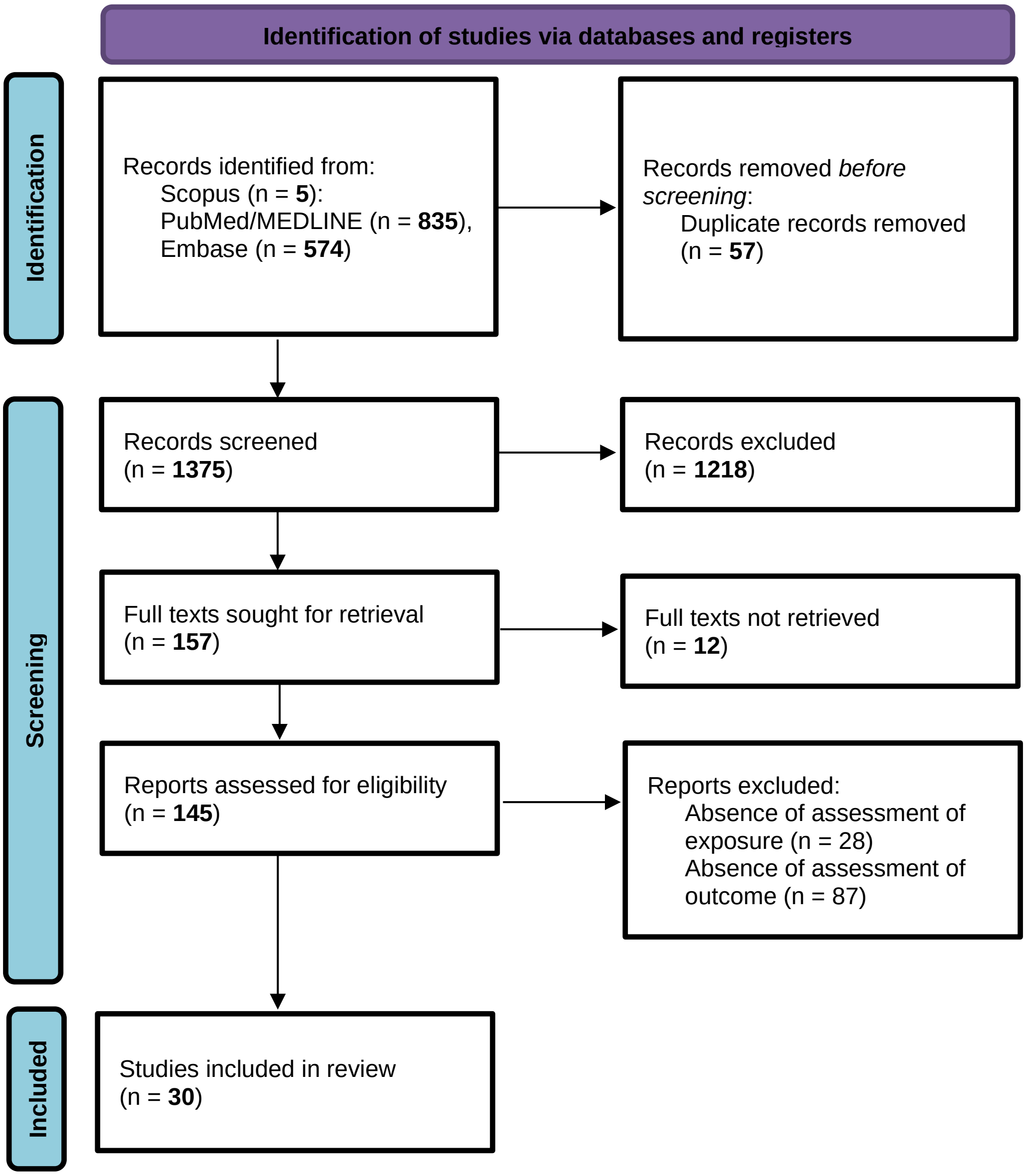

*Consider, if feasible to do so, reporting the number of records identified from each database or register searched (rather than the total number across all databases/registers).

**If automation tools were used, indicate how many records were excluded by a human and how many were excluded by automation tools.

From: Page MJ, McKenzie JE, Bossuyt PM, Boutron I, Hoffmann TC, Mulrow CD, et al. The PRISMA 2020 statement: an updated guideline for reporting systematic reviews. BMJ 2021;372:n71. doi: 10.1136/bmj.n71

For more information, visit: http://www.prisma-statement.org/
