# Supplementary figures and images for: Reinfection incidence following surgical intervention for infected aortic bypass: a meta-analysis
Source: Eur J Clin Microbiol Infect Dis. 2025 Nov 8;45(2):351–62. doi: 10.1007/s10096-025-05248-9 (PMC12987887; doi:10.1007/s10096-025-05248-9)

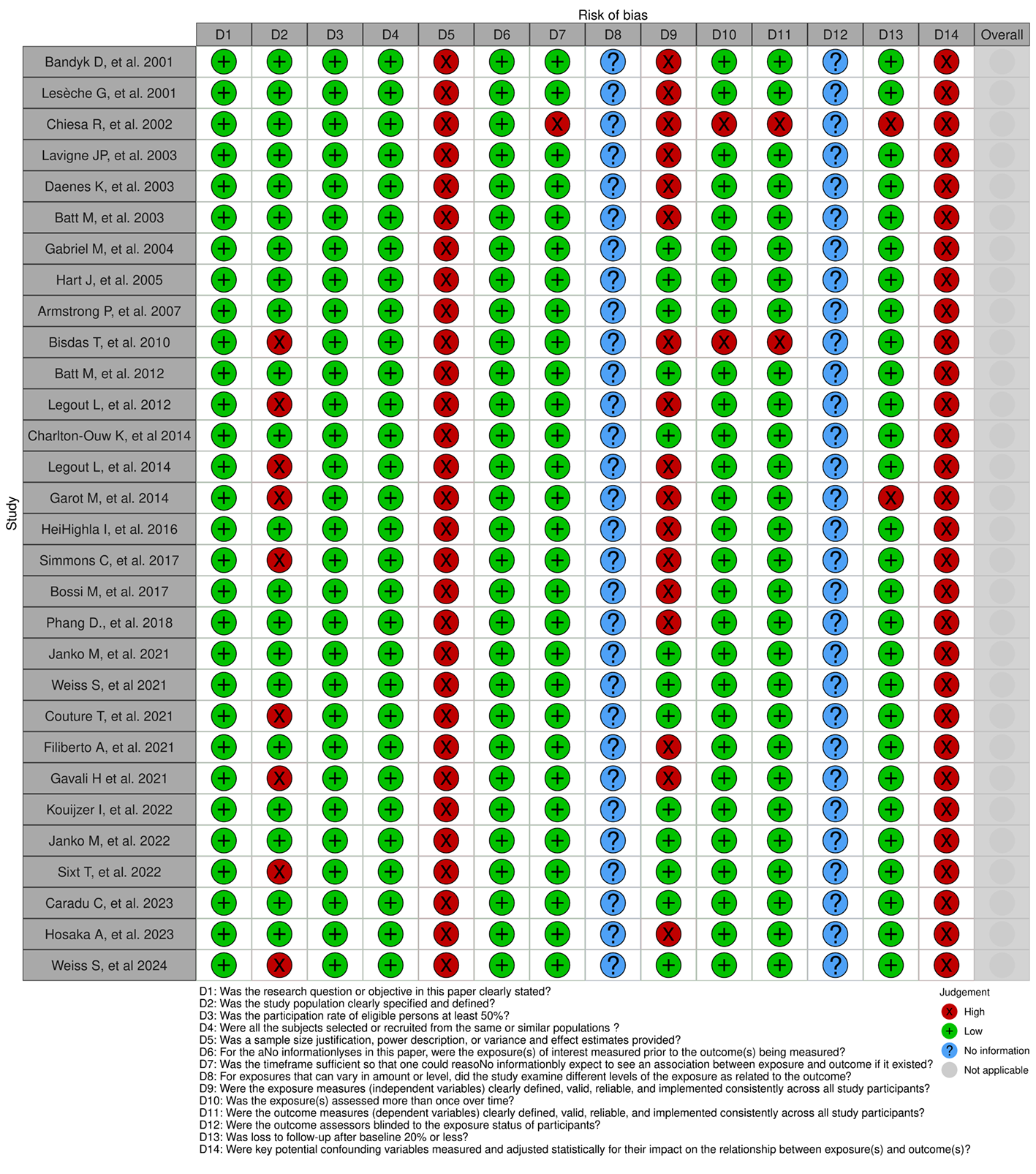

Supplement: Supplementary file 4 — Supplemental Figure 2A (PNG 266 KB) [file 10096_2025_5248_Fig2_ESM.png]

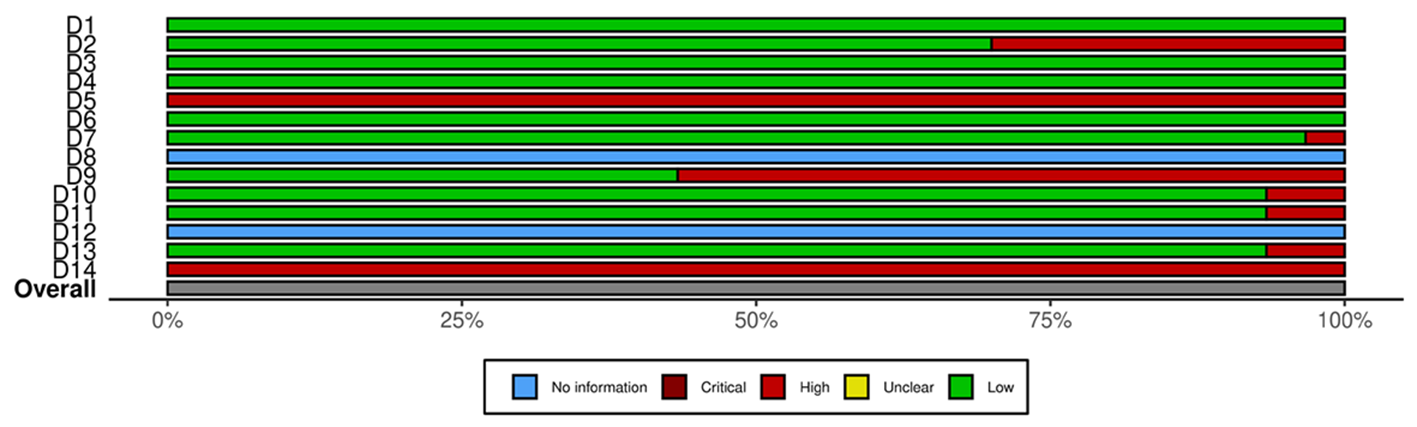

Supplement: Supplementary file 6 — Supplemental Figure 2B (PNG 266 KB) [file 10096_2025_5248_Fig3_ESM.png]

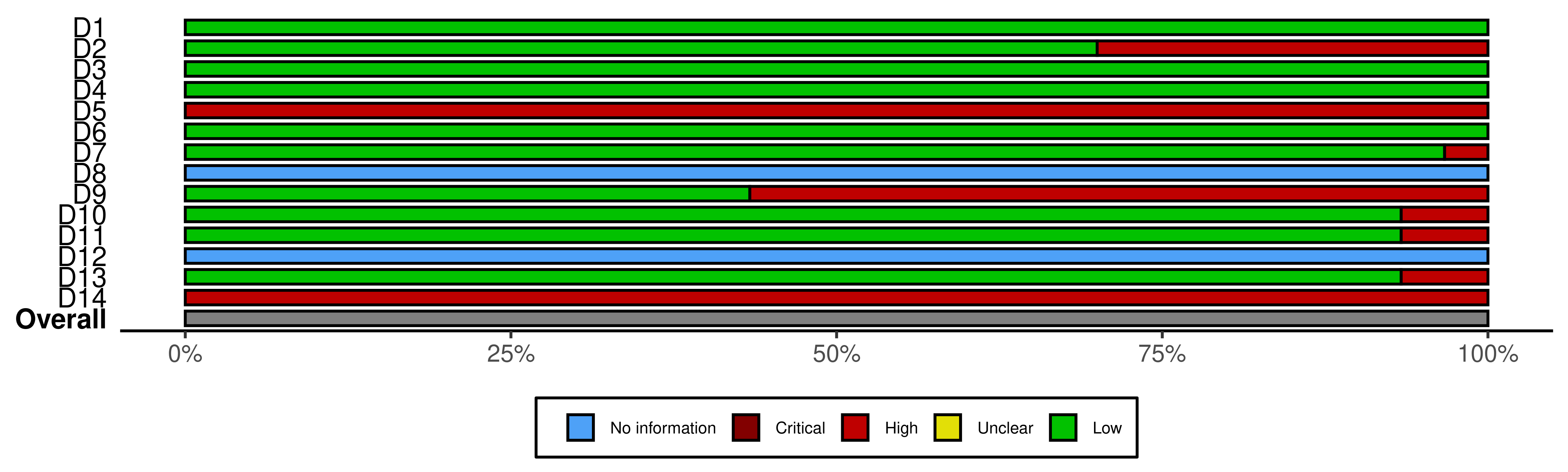

Supplement: Supplementary file 7 — High Resolution Image (TIF 466 KB) [file 10096_2025_5248_MOESM5_ESM.tif]

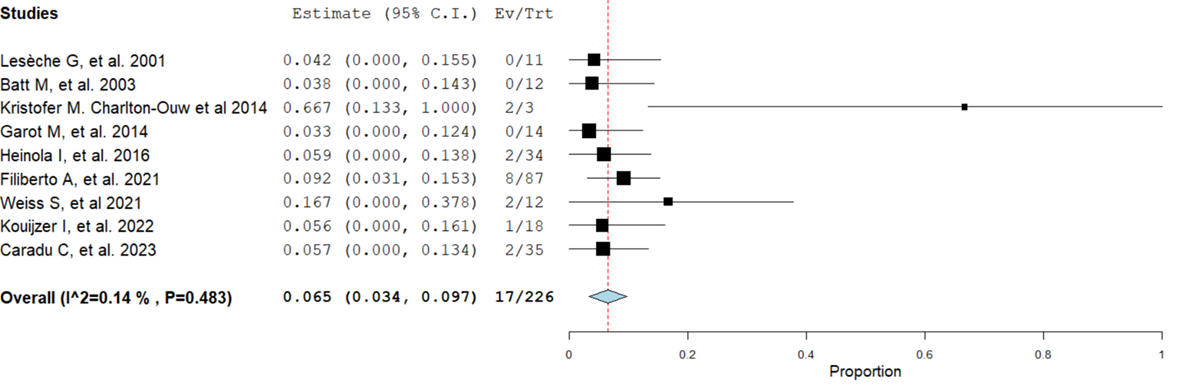

Supplement: Supplementary file 8 — Supplemental Figure 3A (PNG 266 KB) [file 10096_2025_5248_Fig4_ESM.png]

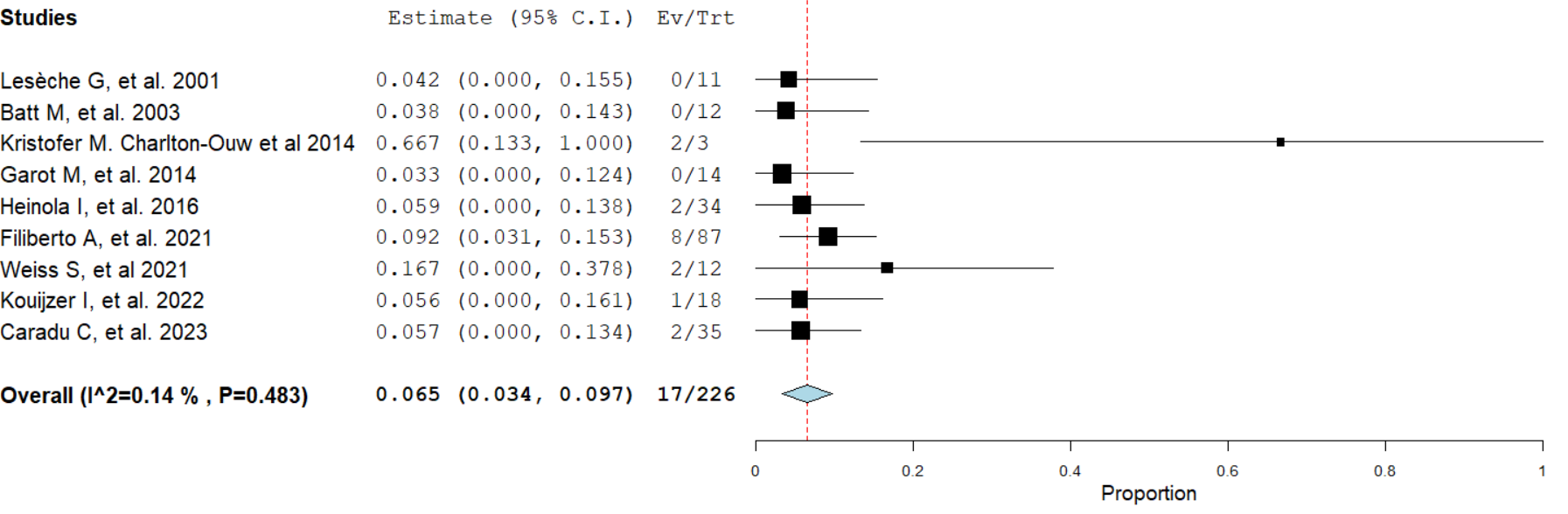

Supplement: Supplementary file 9 — High Resolution Image (TIF 331 MB) [file 10096_2025_5248_MOESM6_ESM.tif]

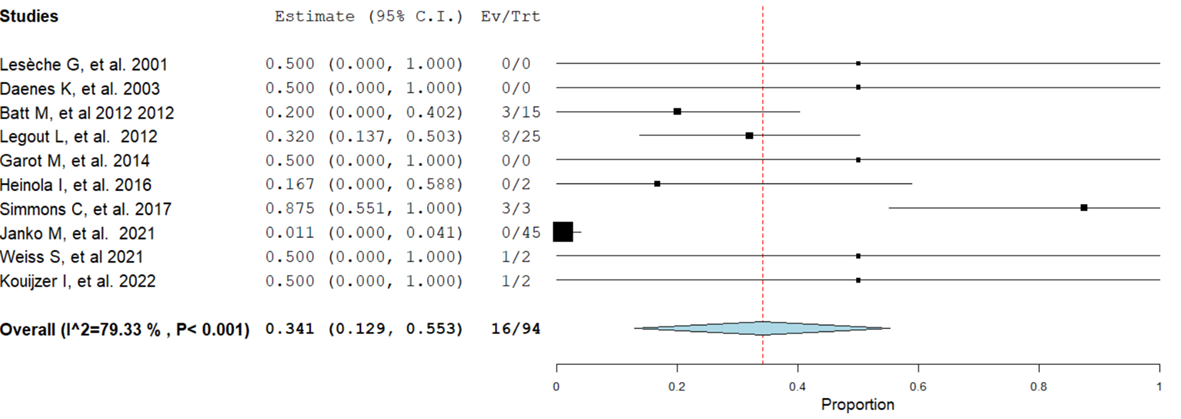

Supplement: Supplementary file 10 — Supplemental Figure 3B (PNG 266 KB) [file 10096_2025_5248_Fig5_ESM.png]

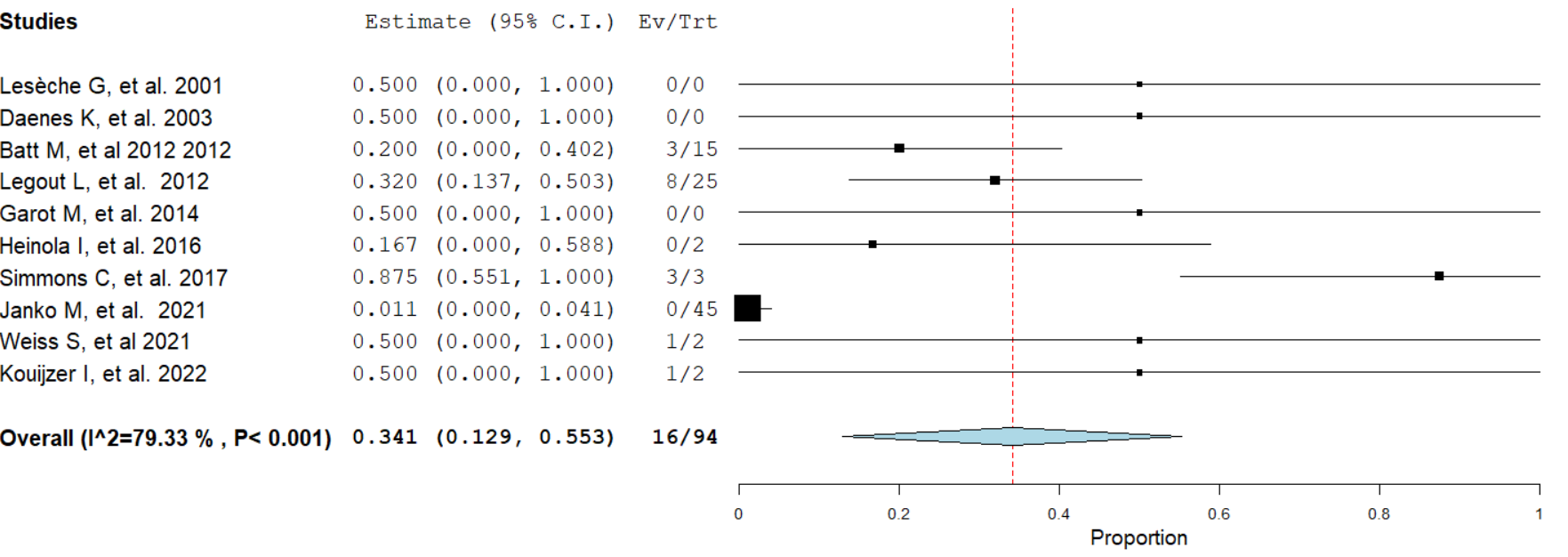

Supplement: Supplementary file 11 — High Resolution Image (TIF 379 KB) [file 10096_2025_5248_MOESM7_ESM.tif]

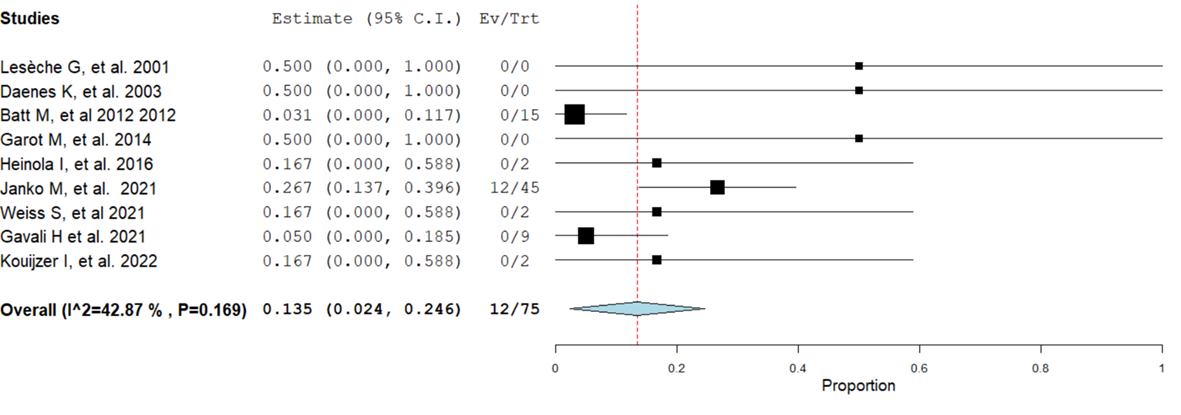

Supplement: Supplementary file 12 — Supplemental Figure 3C (PNG 266 KB) [file 10096_2025_5248_Fig6_ESM.png]

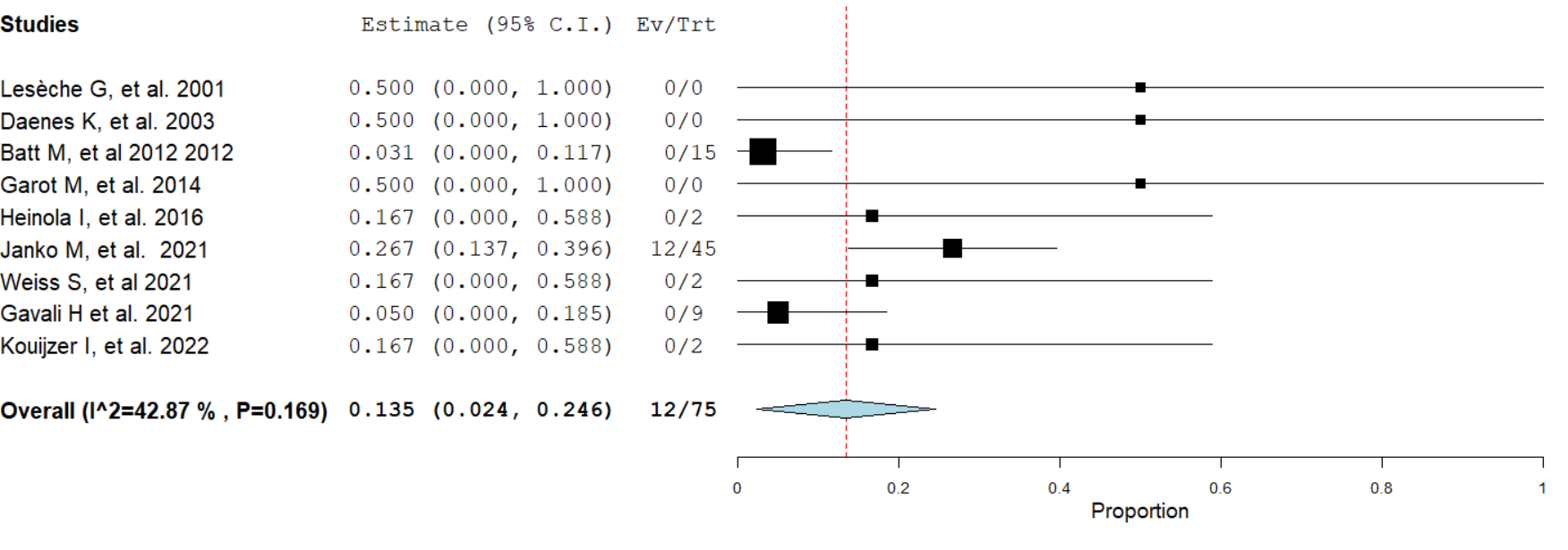

Supplement: Supplementary file 13 — High Resolution Image (TIF 325 KB) [file 10096_2025_5248_MOESM8_ESM.tif]

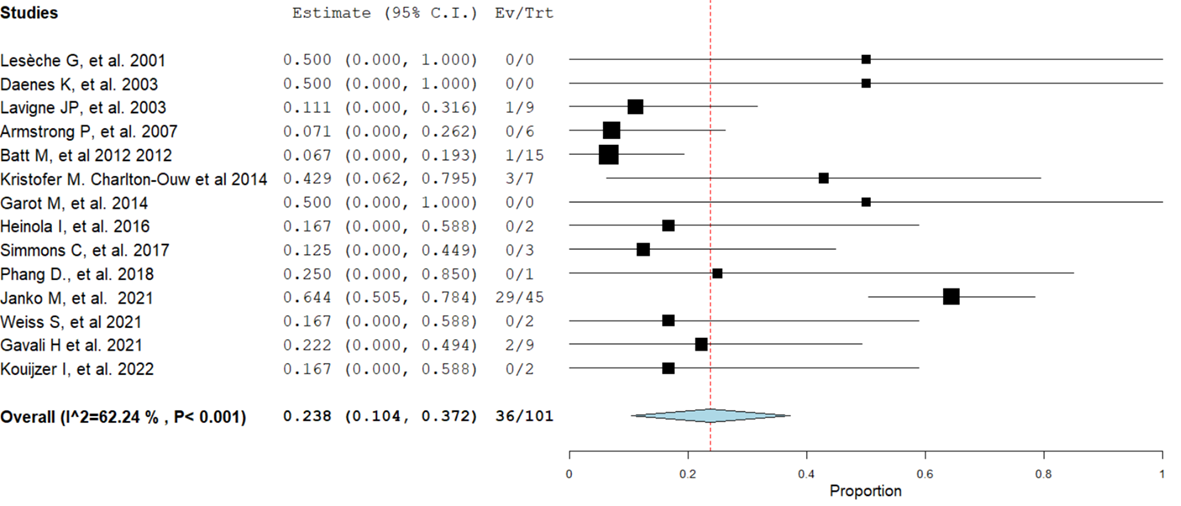

Supplement: Supplementary file 14 — Supplemental Figure 3D (PNG 266 KB) [file 10096_2025_5248_Fig7_ESM.png]

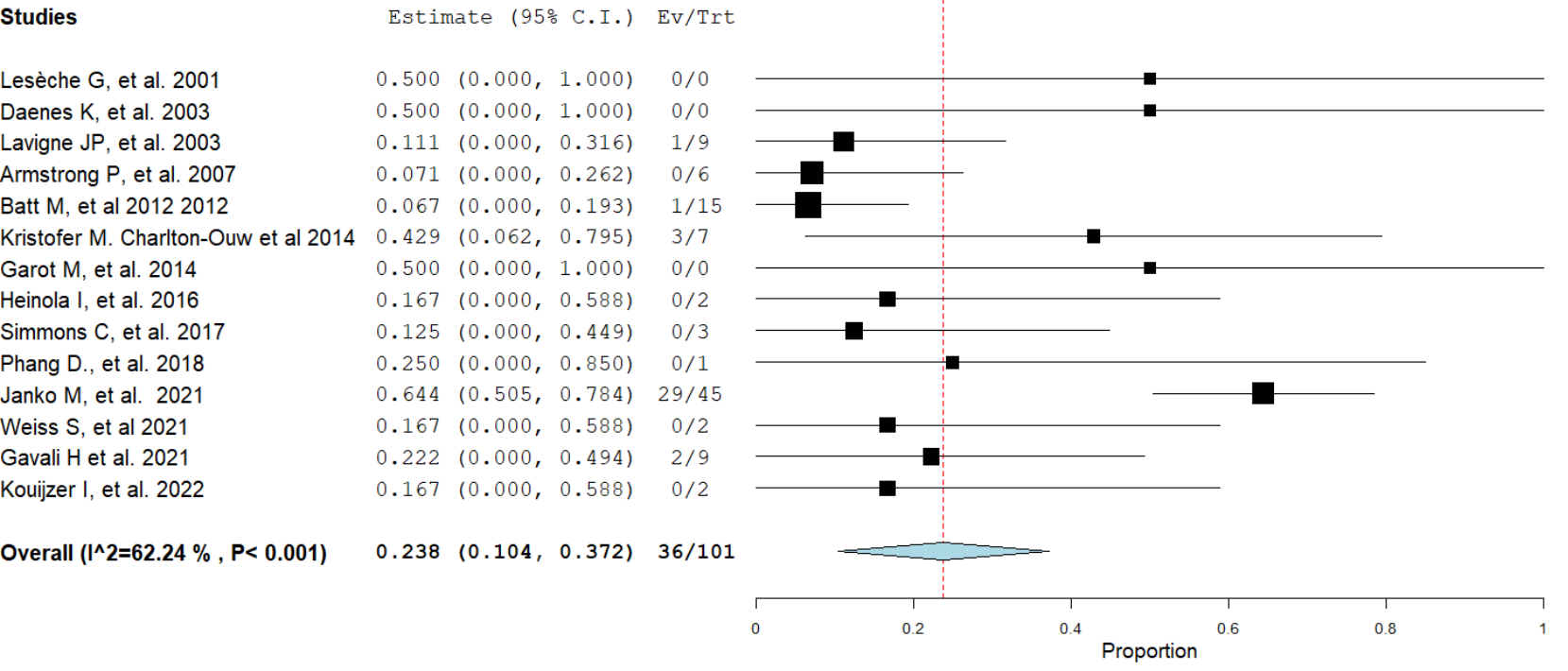

Supplement: Supplementary file 15 — High Resolution Image (TIF 448 KB) [file 10096_2025_5248_MOESM9_ESM.tif]

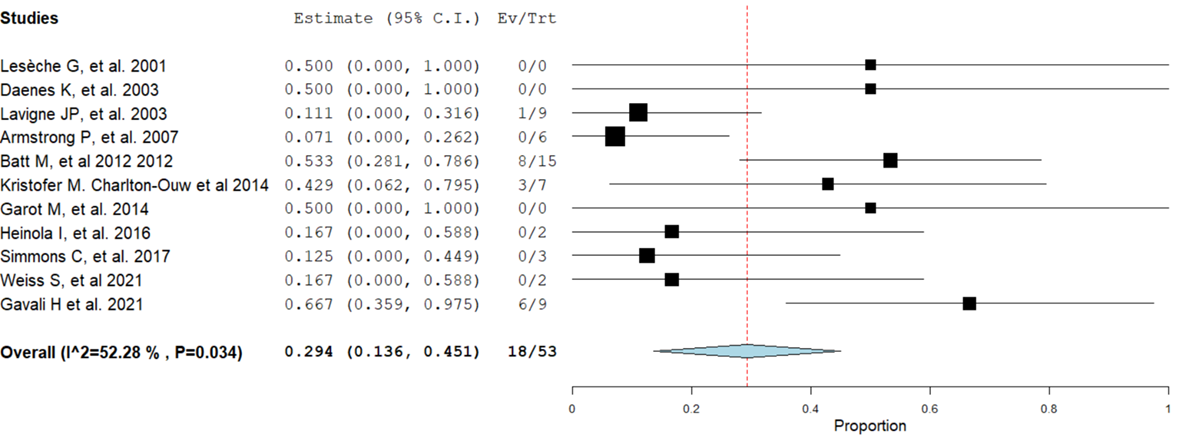

Supplement: Supplementary file 16 — Supplemental Figure 3E (PNG 266 KB) [file 10096_2025_5248_Fig8_ESM.png]

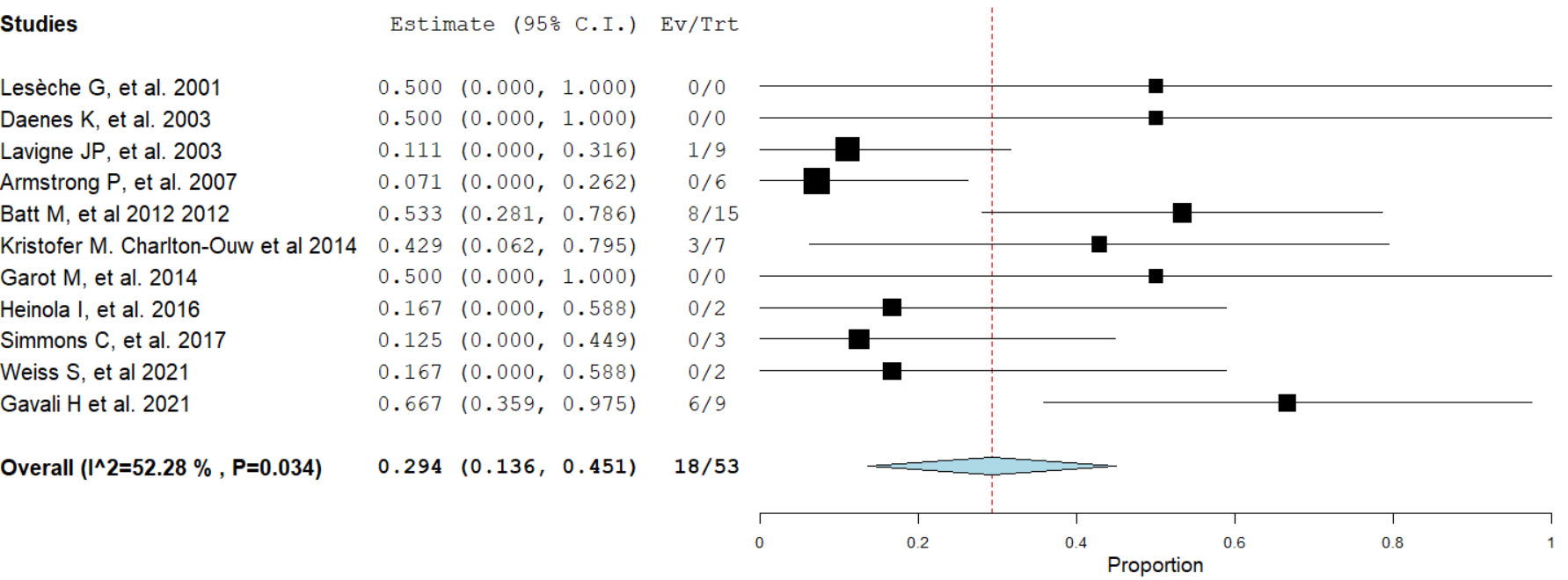

Supplement: Supplementary file 17 — High Resolution Image (TIF 387 KB) [file 10096_2025_5248_MOESM10_ESM.tif]

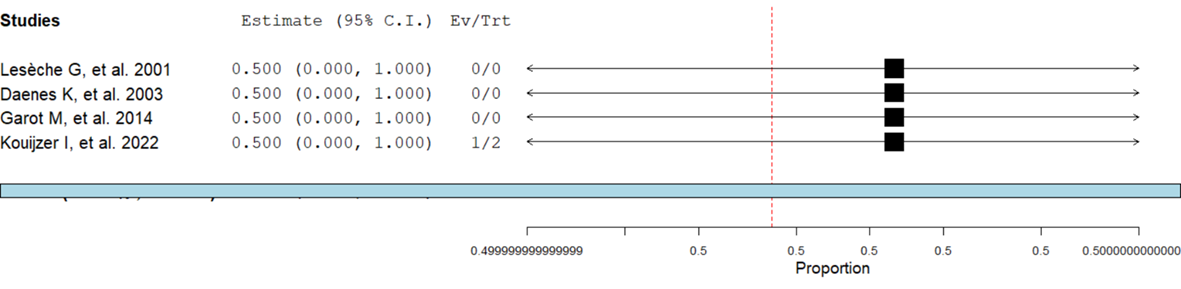

Supplement: Supplementary file 18 — Supplemental Figure 3F (PNG 266 KB) [file 10096_2025_5248_Fig9_ESM.png]

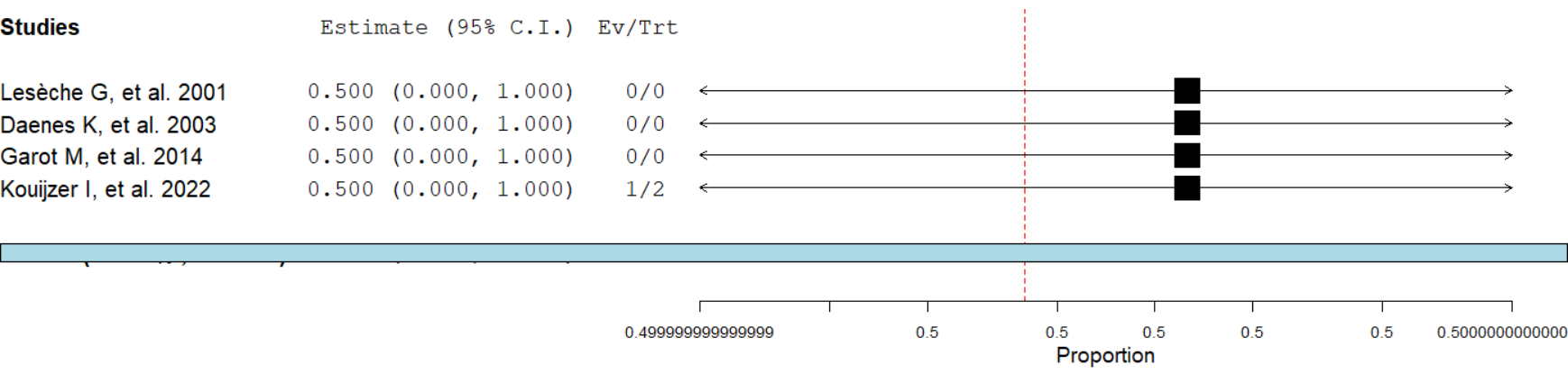

Supplement: Supplementary file 19 — High Resolution Image (TIF 197 KB) [file 10096_2025_5248_MOESM11_ESM.tif]

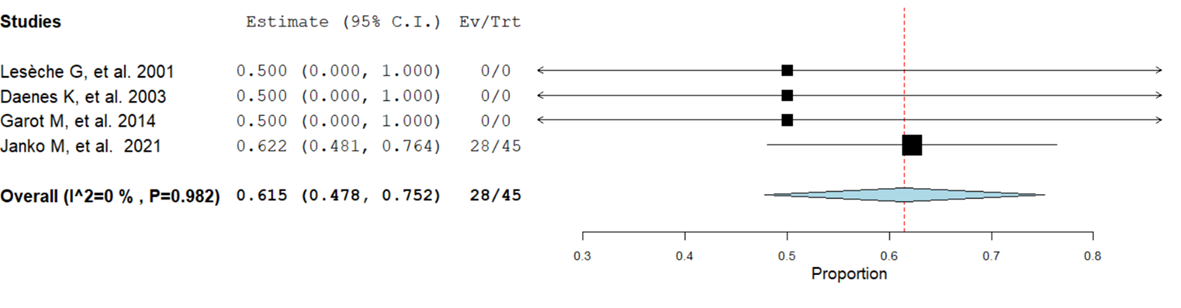

Supplement: Supplementary file 20 — Supplemental Figure 3G (PNG 266 KB) [file 10096_2025_5248_Fig10_ESM.png]

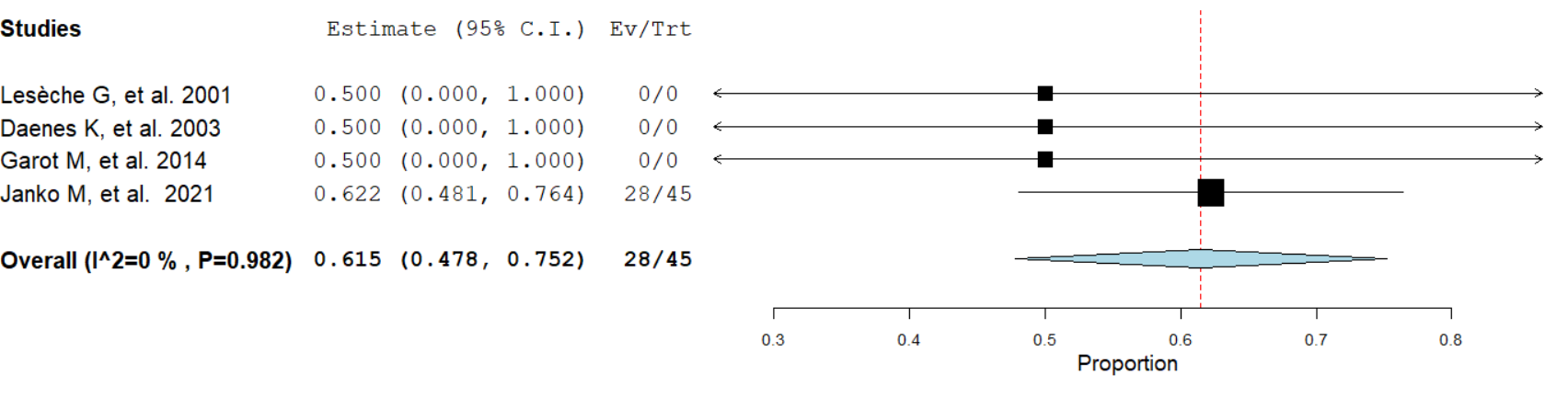

Supplement: Supplementary file 21 — High Resolution Image (TIF 218 KB) [file 10096_2025_5248_MOESM12_ESM.tif]

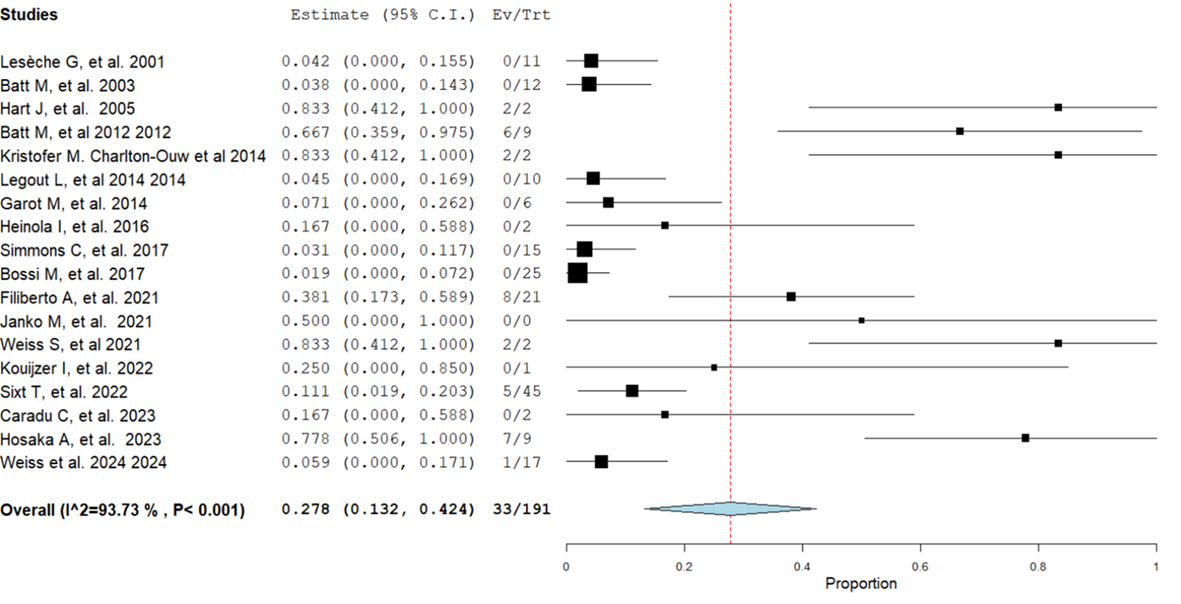

Supplement: Supplementary file 22 — Supplemental Figure 3H (PNG 266 KB) [file 10096_2025_5248_Fig11_ESM.png]

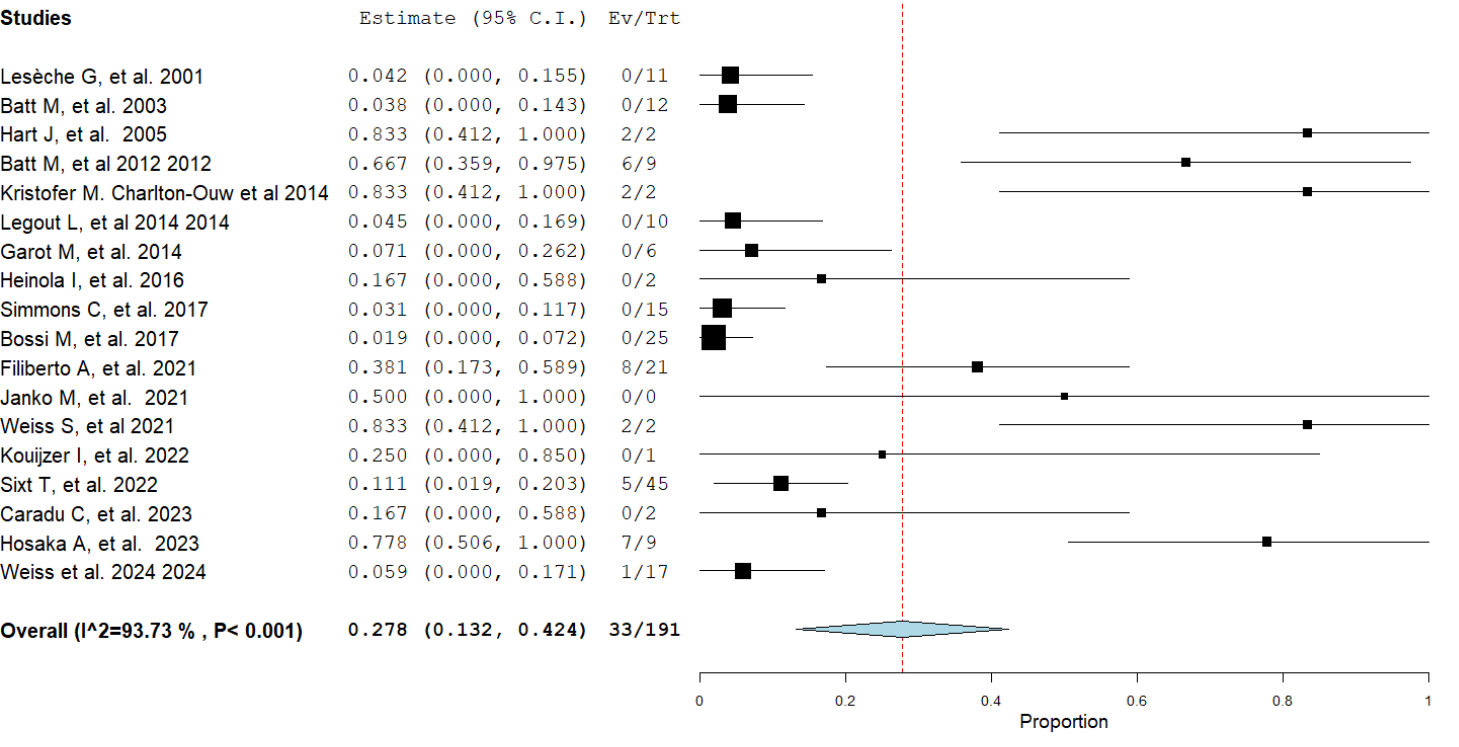

Supplement: Supplementary file 23 — High Resolution Image (TIF 397 KB) [file 10096_2025_5248_MOESM13_ESM.tif]
